# Disulfidptosis-related gene signatures as prognostic biomarkers and predictors of immunotherapy response in HNSCC

**DOI:** 10.3389/fimmu.2024.1456649

**Published:** 2025-01-17

**Authors:** Haotian Qin, Juan Xu, Yaohang Yue, Meiling Chen, Zheng Zhang, Panpan Xu, Yan Zheng, Hui Zeng, Jian Weng, Jun Yang, Fei Yu

**Affiliations:** ^1^ Department of Bone and Joint Surgery, Peking University Shenzhen Hospital, Shenzhen, Guangdong, China; ^2^ Shenzhen Key Laboratory of Orthopaedic Diseases and Biomaterials Research, Peking University Shenzhen Hospital, Shenzhen, Guangdong, China; ^3^ Department of Oncology, Chaohu Hospital of Anhui Medical University, Hefei, China; ^4^ Operating Room, Peking University Shenzhen Hospital, Shenzhen, China; ^5^ Stomatological Center, Peking University Shenzhen Hospital, Shenzhen, China; ^6^ Department of Otolaryngology Head and Neck Surgery, Chaohu Hospital of Anhui Medical University, Hefei, China; ^7^ Department of Pathology, Chaohu Hospital of Anhui Medical University, Hefei, China; ^8^ Department of Orthopedics, Medical Innovation Technology Transformation Center of Shenzhen Second People’s Hospital, Shenzhen Second People’s Hospital, Shenzhen, Guangdong, China; ^9^ Department of Radiology, Peking University Shenzhen Hospital, Shenzhen, China; ^10^ Department of Spine Surgery, Shenzhen Second People’s Hospital, The First Affiliated Hospital of Shenzhen University, Shenzhen, China

**Keywords:** disulfidptosis, head and neck squamous cell carcinoma, prognostic signatures, bioinformatics analysis, immunotherapy response

## Abstract

**Background:**

Disulfidptosis is a newly discovered form of cell death associated with tumorigenesis, particularly under oxidative stress and metabolic disorder conditions. Currently, the biological mechanisms of disulfidptosis-related genes (DRGs) in head and neck squamous cell carcinoma (HNSCC) remain unclear.

**Methods:**

The study includes sections on methodologies, data sources, clinical data collection, subtype establishment, identification and analysis of differentially expressed genes, genetic variation, and the construction and validation of a DRG prognostic model. Various analyses are conducted, including the relationship between the risk scores model and clinicopathological features, immune status, immune checkpoints, tumor mutational burden (TMB), microsatellite instability (MSI), ESTIMATE, mRNAsi, and drug sensitivity. The study also covers single-cell analysis and DNA methylation analysis of DRGs, and the prediction of potential microRNA and long non-coding RNA target genes. Prognostic DRGs expression in HNSCC is validated through RT-qPCR and immunohistochemistry. The model’s predictive capability is confirmed using external validation cohorts from GEO datasets and clinical tissue samples. The role of DSTN in HNSCC is further validated through gene knockout experiments.

**Results:**

We identified four valuable genes (SLC3A2, NUBPL, ACTB, DSTN) and constructed a prognostic model, along with identifying two DRG-related subtypes. Analysis of the DRG risk score revealed that the low-risk group had a better prognosis compared to the high-risk group. Significant correlations were found between the DRG risk score and clinical features, immunotherapy response, drug sensitivity, and genes related to RNA epigenetic modifications. Low-risk HNSCC patients were identified as potential beneficiaries of immune checkpoint inhibitor (ICI) therapy. A regulatory axis involving DSTN, hsa-miR-181c-5p, LUCAT1, and IGFL2-AS1 was constructed for HNSCC. RT-qPCR and IHC data further validated the upregulation of prognostic DRGs in HNSCC. The prognostic model demonstrated excellent predictive performance for the prognosis of HNSCC patients. Additionally, DSTN was significantly overexpressed in tumor cells; its knockdown inhibited tumor cell proliferation, migration, and invasion.

**Conclusion:**

The prognostic model effectively predicts HNSCC outcomes, with better prognosis in the low-risk group. DSTN upregulation promotes tumor growth, and its knockout inhibits proliferation, migration, and invasion.

## 1 Introduction

Head and neck squamous cell carcinoma (HNSCC) is the sixth most common cancer globally, encompassing malignant tumors in the oral cavity, nasal cavity, pharynx, larynx, neck, and upper esophagus. Over 90% of cases are squamous cell carcinomas, making HNSCC one of the predominant pathological types of cancer originating in the head and neck region (1). The clinical prognosis of HNSCC patients is influenced by various factors, including tumor size, location, the patient’s overall health, and the tumor’s biological characteristics (2). Most HNSCC patients are diagnosed at an advanced stage, with high rates of local recurrence and lymph node metastasis, resulting in a low overall survival rate (3, 4). Despite advancements in treatment methods in recent years, the long-term survival rate of HNSCC patients has seen limited improvement. Consequently, identifying new biomarkers to better understand tumor behavior and predict treatment responses has become a research focus.

Disulfidptosis, a recently discovered form of cell death characterized by abnormally elevated levels of intracellular sulfides, is particularly prevalent in cancer cells due to their aberrant metabolic pathways and stress response mechanisms (5, 6). In solid tumors such as HNSCC, disulfidptosis may be related to tumorigenesis, progression, and response to treatment (7). Recent studies have suggested that disulfidptosis is associated with immune modulation within the tumor microenvironment, potentially influencing tumor response to therapies, including immune checkpoint inhibitors (8). Notably, recent bioinformatics analyses have shed light on the roles of disulfidptosis-related genes (DRGs) in head and neck squamous cell carcinoma (HNSCC), suggesting their potential as predictive biomarkers for prognosis and treatment response. For instance, several studies have demonstrated that DRGs can influence immune cell infiltration and the tumor immune landscape, both of which are pivotal in determining the efficacy of immunotherapies in HNSCC patients (9). Similarly, other researchers have examined the relationship between DRGs and tumor progression in HNSCC using large-scale genomic data, providing valuable insights into how these genes contribute to immune evasion and therapeutic resistance (10). Furthermore, additional studies have elucidated the molecular mechanisms through which DRGs regulate tumor progression, highlighting their roles in modulating cell death pathways and immune cell functions within the tumor microenvironment (11).

The background of this study is based on a comprehensive genomic analysis of HNSCC patient cohorts, aiming to develop a set of predictive DRG prognostic signatures. These signatures can forecast not only clinical outcomes but also patient responses to immune checkpoint inhibitors. We performed an in-depth analysis of HNSCC patient samples using various public databases, including The Cancer Genome Atlas (TCGA) and the Gene Expression Omnibus (GEO). By comparing patient groups with varying survival times, we identified a series of DRGs associated with differential prognoses. Further mechanistic studies revealed how these genes regulate tumor cell death and affect the function of immune cells within the tumor microenvironment. Additionally, we evaluated the effectiveness of these genes in predicting patient responses to immune checkpoint inhibitors. Our preliminary results indicate that these DRGs are associated with overall survival rates, immune-related gene expression, the abundance of tumor-infiltrating lymphocytes, and responses to immune checkpoint inhibitors. These findings provide insights into developing new therapeutic strategies, particularly for patients who do not respond to existing immunotherapies.

In summary, this paper highlights the significance of disulfidptosis in HNSCC treatment, especially in assessing clinical prognosis and immunotherapy response. Although this field is still in its early stages, its potential in personalized medicine and precision treatment cannot be overlooked. As future research progresses, disulfidptosis is expected to become a key factor in improving treatment outcomes for HNSCC patients.

## 2 Materials and methods

### 2.1 Data sources and preprocessing

This study utilized RNAseq data and corresponding clinical information for HNSCC from The Cancer Genome Atlas (TCGA) (https://portal.gdc.cancer.gov/) (12). The dataset included 504 HNSCC patients and 44 normal tissue samples. All data were standardized per million transcripts (Transcripts Per Million, TPM) and normalized to approximate a normal distribution using the R software package “ggplot2” (v4.0.3). Gene expression data were extracted to construct a data matrix and analyzed using the Wilcoxon test.

### 2.2 Clinical data and tissue sample collection

Clinical data and tissue samples were collected from Chaohu Hospital of Anhui Medical University and Peking University Shenzhen Hospital. The study included 76 HNSCC patients admitted between September 2016 and September 2018. Paraffin-embedded pathological sections of HNSCC tissues and adjacent normal tissues (0.5 cm) were collected, along with complete clinical case data and follow-up information. Among the patients, 56 were male and 20 were female, aged between 35 and 87 years (mean age 62.737 ± 10.836 years), with a median age of 66.0 years. Overall survival (OS) was defined as the period from the date of surgery to the date of death or last follow-up. Follow-up was conducted monthly for the first 3 months, every 3 months for 2 years, every 6 months for the next 3 years, and annually thereafter, ending in September 2023. Survival times ranged from 1.22 to 60 months, with a median survival time of 51.51 months (interquartile range: 19.427 to 60.0 months). All patients were confirmed by pathological examination, and tumor TNM staging was evaluated using the 8th edition of the American Joint Committee on Cancer (AJCC) staging system. The use of HNSCC samples was approved by the Ethics Committee of Chaohu Hospital of Anhui Medical University (approval No. KYXM202310004) and the Ethics Committee of Peking University Shenzhen Hospital (approval No. 2022-117). The study was conducted in accordance with the Declaration of Helsinki (as revised in 2013). All patients provided written informed consent.

### 2.3 Establishment of subtypes

Based on previous literature, we identified 24 potential disulfidptosis-related genes (DRGs) (6) (**Supplementary Table 1**). Using the consistent clustering of these 24 genes, we performed consistency analysis with the R package “ConsensusClusterPlus” (v1.54.0) (13). The maximum number of clusters was set to 6 (k=6), and 80% of the total sample was drawn 100 times, with clusterAlg = “hc” and innerLinkage=‘ward.D2’. The number of clusters varied from 2 to 6 (k=2-6), and the consistency matrix and the consistency cumulative distribution function (CDF) were evaluated to determine the best classification. Clustering heat maps were generated using the R package “pheatmap” (v1.0.12). Gene expression heat maps retained motifs with a variance above 0.1. Based on the expression profiles of DRGs, TCGA cases were divided into Cluster1 (C1) and Cluster2 (C2).

### 2.4 Identification and enrichment analysis of differentially expressed genes

Differentially expressed genes (DEGs) between C1 and C2 subtypes were identified using the Limma package (v3.40.2) (14) in R software. The adjusted P value was analyzed in the TCGA database to correct for false positives. “Adjusted P < 0.05 and log2 (Fold change) > 1.5 or log2 (Fold change) < -1.5” was defined as the standard for screening differential expression of mRNA. Heat maps were generated using the R software heatmap package. The Gene Ontology (GO) function of DEGs and their enrichment in the Kyoto Encyclopedia of Genes and Genomes (KEGG) pathway were analyzed using the R package “clusterProfiler” (v3.18.0) (15). Additionally, gene set enrichment analysis (GSEA) (http://software.broadinstitute.org/gsea/index.jsp) (16) was employed to identify potential biological pathways. DEGs from TCGA data were categorized into up-regulated and down-regulated groups. In each analysis, 10,000 gene combinations were tested to identify pathways with significant changes. Genes were considered enriched in meaningful pathways when p.adjust < 0.05 and FDR (false discovery rate) < 0.25.

### 2.5 Genetic variation

Gene Set Cancer Analysis (GSCA) (http://bioinfo.life.hust.edu.cn/GSCA/#/) (17) integrated expression, mutation, drug sensitivity, and clinical data from four public data sources for 33 cancer types. Somatic mutations of HNSCC patients were downloaded and visualized using the maftools package in R software, encompassing seven types of mutations: Missense_Mutation, Splice_Site, Nonsense_Mutation, Frame_Shift_Del, Frame_Shift_Ins, In_Frame_Del, Multi_Hit. This study also analyzed the Spearman correlation between the expression of DRGs mRNA and Copy Number Variation (CNV), and methylation. We investigated the correlation between methylation, CNV, and survival outcomes in HNSCC patients, including Disease-Free Interval (DFI), Disease-Specific Survival (DSS), Overall Survival (OS), and Progression-Free Survival (PFS).

### 2.6 Construction and validation of DRG prognostic model

Based on the levels of the aforementioned DRGs associated with HNSCC prognosis, LASSO-Cox regression analysis was performed to construct the prognostic model. According to the results of multivariate Cox regression analysis, the prognostic DRGs risk score was calculated as follows: Riskscore = ∑i Coefficient (mRNAi) × Expression (mRNAi). The entire TCGA-HNSCC dataset was used as the training cohort, and patients were divided into low-risk and high-risk subtypes based on the average risk score. The overall survival rates of the two subtypes were compared using Kaplan–Meier analysis, and time ROC analysis was conducted to predict the model’s accuracy. The optimal truncated expression value was determined using the “surve_cutpoint” function of the “survminer” R package. The validation cohort was then used to verify the accuracy of the DRGs signature with the GSE41613, GSE65858, GSE85446 datasets and clinical HNSCC tissue samples (n=76) serving as the external validation cohort, further corroborating the results.

### 2.7 Relationship between DRGs and clinicopathological features and prognosis in HNSCC

Using the log-rank test and univariate Cox regression analysis, Kaplan–Meier curves, P values, and hazard ratios (HRs) with 95% confidence intervals (CIs) were obtained. Subsequently, key prognostic DRGs (SLC3A2, UNBPL, ACTB, and DSTN) in HNSCC patients were identified and analyzed in detail. The relationship between prognosis-related DRGs and the overall survival rate of HNSCC patients was examined, and the area under the receiver operating characteristic (ROC) curve was calculated. The expression and diagnostic efficacy of DRGs in HNSCC were validated using datasets obtained from NCBI-GEO (https://www.ncbi.nlm.nih.gov/gds) (18), including 184 HNSCC tissues and 45 para-cancerous tissues from GSE30784 and 18 HNSCC tissues and 18 para-cancerous tissues from GSE53819. Additionally, we analyzed the prognostic outcomes between high and low-risk groups across different clinical subgroups. Clinicopathological data of HNSCC patients, including age, sex, race, T, N, M, stage, grade, smoking, radiation, and neoadjuvant therapy, were obtained from TCGA.

### 2.8 Building and validation of a predictive nomogram

The “rms” package was utilized to construct a nomogram model for predicting 1-, 3-, and 5-year OS, PFS, and DSS based on the results of multivariate Cox proportional hazards analysis. The calibration curve and decision curve analysis (DCA) were used to validate the model’s predictive performance. External validation was performed using the GSE65858 dataset and clinical HNSCC tissue samples to evaluate the prediction model’s accuracy.

### 2.9 Analysis of gene expression related to immune infiltration and immune checkpoints

For immune scoring, the R software immunedeconv package (19) and six advanced algorithms, including TIMER (20), xCell (21), MCP-counter (22), CIBERSORT (23), EPIC (24), and quantTIseq (25), were used to compare the degree of immune cell infiltration between C1and C2 subtypes via the Wilcoxon test. Additionally, single-sample gene set enrichment analysis (ssGSEA) in the R package “GSVA” (26) was used to quantify the infiltration levels of various immune cell types. The infiltration and accumulation of 23 common immune cells, including dendritic cells (DC), immature DC (iDC), activated DC (aDC), plasmacytoid DC (pDC), T helper (Th) cells, type 1 Th cells (Th1), type 2 Th cells (Th2), type 17 Th cells (Th17), regulatory T cells (Treg), T gamma delta (Tgd), T central memory (Tcm), T effector memory (Tem), T follicular helper (Tfh), CD8+ T cells, B cells, neutrophils, macrophages, cytotoxic cells, mast cells, eosinophils, natural killer (NK) cells, NK56- cells, and NK56+ cells, were analyzed. The Wilcoxon rank-sum test was performed to compare differences in immune cell infiltration levels of the four prognosis-related DRGs between high and low expression groups and between high-risk and low-risk groups. The correlation between immune cell infiltration and prognosis of HNSCC patients was also investigated. Spearman correlation was used to explore the relationship between the four prognosis-related DRGs and immune cell infiltration. TIMER (https://cistrome.shinyapps.io/timer/) was used to analyze the abundance of immune cells infiltrated by the four prognostic DRGs in tumors. The detected immune cells included tumor purity, B cells, CD4+ T cells, CD8+ T cells, neutrophils, macrophages and dendritic cells. Immune cell abundance (immune score), stromal cell infiltration level (stromal score), and tumor purity (ESTIMATE score) were estimated using the ESTIMATE algorithm. The expression levels of several immune checkpoint-related genes (CD274, CTLA4, HAVCR2, LAG3, PDCD1, PDCD1LG2, TIGIT, and SIGLEC15) were analyzed between C1 (N=461) and C2 (N=43) subtypes and between high-risk and low-risk groups. Spearman correlation was used to explore the association between risk scores and immune checkpoint-related genes. The Tumor Immune Dysfunction and Exclusion (TIDE) algorithm was used to predict potential immune checkpoint blocking responses. The results were visualized using the R packages “ggplot2” and “pheatmap” (v4.0.3) (27).

### 2.10 TMB, MSI, mRNAsi, and drug sensitivity analysis

The correlation of the risk score in HNSCC with tumor mutation burden (TMB), microsatellite instability (MSI), and mRNA stemness index (mRNAsi) was analyzed using Spearman correlation. The sensitivity of these drugs was also studied. Drug sensitivity and gene expression profile data from cancer cell lines were integrated from the Drug Sensitivity in Cancer (GDSC) (https://www.cancerrxgene.org/) (28) and the Cancer Therapeutics Response Portal (CTRP) (https://portals.broadinstitute.org/ctrp/) databases. The 50% inhibiting concentration (IC50) of chemotherapeutic drugs was predicted using the R package pRRophetic (29), with the IC50 value of the sample estimated by ridge regression. All parameters were set to default values, the batch effect was adjusted using combat, and the tissue type was considered. Duplicate gene expression was summarized as the mean value.

### 2.11 Single cell analysis

The expression of DRGs in the tumor microenvironment (TME) was studied using the Tumor Immune Single Cell Center (TISCH) (http://tisch.comp-genomics.org/) (30) to understand their relationship with HNSCC prognosis. In this dataset, three main cell types were included: immune cells, stromal cells, and malignant cells. The t-distributed stochastic neighborhood embedding (t-SNE) map of HNSCC_GSE103322 and the heat map of HNSCC_GSE103322 were displayed through the TISCH database to show the impact of DRGs on the TME in HNSCC. Additionally, scatter plots showing the correlation between DRG immune infiltration levels and cancer-associated fibroblasts (CAFs) and macrophages were generated using TIMER2.0 (http://timer.cistrome.org/) (31).

### 2.12 DNA methylation analysis of DRGs in HNSCC

The GSCA database was used to evaluate the relationship between the expression of four prognostic DRGs and DNA methylation levels. NUBPL methylation levels were measured in HNSCC patients grouped by different clinicopathologic features, including age, gender, race, smoking status, nodal metastasis status, tumor grade, individual cancer stage, and TP53 mutation, using the UALCAN database (http://ualcan.path.uab.edu/index.html) (32). The MethSurv database (https://biit.cs.ut.ee/methsurv/) (33) was then used to analyze the DNA methylation of four prognostic DRGs at CpG sites and the prognostic value of these CpG methylation sites in HNSCC.

### 2.13 Relationship between DRG expression level and RNA modification regulatory factors

Using the Wilcoxon test and the “ggplot2” package in R software (v4.0.3), differences in gene expression between high and low-risk groups for m6A, m5C, m1A, and m7G genes in HNSCC samples were analyzed. The correlation between the risk score in HNSCC samples and the expression of m6A, m5C, m1A, and m7G genes was also examined. The expression matrix for m6A genes includes RBM15B, VIRMA, IGF2BP2, HNRNPA2B1, IGF2BP1, YTHDF3, IGF2BP3, HNRNPC, RBM15, RBMX, METTL14, YTHDC2, METTL3, ZC3H13, WTAP, YTHDF1, YTHDC1, FTO, and YTHDF2. m5C genes include DNMT1, DNMT3A, DNMT3B, MBD1, MBD2, MBD3, MBD4, MECP2, NEIL1, SMUG1, TDG, UHRF1, UHRF2, UNG, ZBTB33, ZBTB38, ZBTB4, TET1, TET2, and TET3. m1A genes include TRMT10C, TRMT61B, TRMT6, TRMT61A, ALKBH3, ALKBH1, YTHDC1, YTHDF1, YTHDF2, and YTHDF3. m7G genes include AGO2, CYFIP1, DCP2, DCPS, EIF3D, EIF4A1, EIF4E, EIF4E2, EIF4E3, EIF4G3, GEMIN5, IFIT5, LARP1, LSM1, METTL1, NCBP1, NCBP2, NCBP2L, NCBP3, NSUN2, NUDT10, NUDT11, NUDT16, NUDT3, NUDT4, SNUPN, and WDR4.

### 2.14 Prediction of potential MicroRNA and long non-coding RNA target genes

ENCORI (http://starbase.sysu.edu.cn/) (34), miRTarBase (https://mirtarbase.cuhk.edu.cn/) (35), RNA22 (https://cm.jefferson.edu/rna22/interactive) (36), RNAInter (http://www.rnasociety.org/rnainter/) (37), and miRWalk (http://miRWalk.umm.uni-heidelberg.de/) (38) databases were used to screen candidate microRNAs (miRNAs) and predict miRNA targets. These selected miRNAs are referred to as potential miRNAs of target genes. The potential combinations of long non-coding RNAs (lncRNAs) and miRNAs were predicted using the miRNet database (http://www.mirnet.ca/) (39). Subsequently, the mRNA-miRNA and miRNA-lncRNA regulatory networks were established using Cytoscape (version 3.7.1; http://www.cytoscape.org/) (40). The correlation and prognostic value of these candidate miRNAs and lncRNAs in HNSCC were further verified using the ENCORI and Kaplan–Meier plotter databases.

### 2.15 Cell culture and transfection

Three HNSCC cell lines (HN6, HSC3, and SCC9) and a human normal squamous cell line (NOK) were used in this study. NOK, HN6, HSC3, and SCC9 cell lines were purchased from the American Type Culture Collection (ATCC; Manassas, VA, USA). HN6 and HSC3 cells were cultured in DMEM (Sigma, USA, D5546), supplemented with 10% FBS (Gibco, 10099-141C) and 1% penicillin-streptomycin solution (Gibco, Massachusetts, USA, 15070063). SCC9 cells were supplemented with 10% FBS (Gibco, 10099-141C), 1% penicillin-streptomycin solution (Gibco, 15070063), and hydrocortisone (1 ng/mL; MCE, HY-N0583). NOK cells were grown in defined keratinocyte-SFM (Gibco, 10744019) supplemented with Defined Keratinocyte-SFM Growth Supplement (Gibco, 10744019) and 1% penicillin-streptomycin solution. All cultures were maintained in a humidified incubator with 5% CO2 at 37°C. After passaging cells and culturing them in a six-well plate for 24 hours, transfection was performed when the cell density reached 60–70%. Transfection of shRNA-DSTN (GeneRulor, Zhuhai) was carried out using Lipofectamine 3000 transfection reagent (Invitrogen, USA). Cells were collected 48 hours post-transfection to extract RNA for assessing transfection efficiency, with all experiments performed in triplicate.

### 2.16 Proliferation and colony formation assay

For the proliferation assay, 2000 cells were seeded in a 96-well plate after the indicated treatment. On the next day, cell viability was detected using the Cell Counting Kit-8 (CCK-8) assay (Dojindo, Japan) according to the manufacturer’s instructions. Each experiment was conducted in triplicate, and cell viability was measured continuously for 5 days. For the colony formation assay, 1000 cells were seeded in a six-well plate with complete medium and grown for approximately 2 weeks. Visible colonies were then fixed with 4% paraformaldehyde, stained with 1% crystal violet, and counted.

### 2.17 Wound healing assays

Cells were seeded in 6-well plates and cultured to 90% confluence. A scratch was made across the plates using a pipette tip, and isolated cells were removed with PBS. Images of the wound were captured after 24 hours of incubation. The wound area was measured using Image J.

### 2.18 Transwell assays

24-well transwell chambers, coated with or without Matrigel (Corning, NY, USA, 354480, 3422), were used to analyze cell migration and invasion. Cells suspended in serum-free culture medium were planted into the upper chamber, while medium containing 10% FBS was added to the bottom chamber as an attractant. After 24 hours of incubation, cells remaining in the upper chamber were wiped off with cotton swabs. Cells that had penetrated the transwell chambers were fixed with methanol and stained with crystal violet. The number of cells in five random fields of view (×100 magnification) was counted under a microscope.

### 2.19 RNA isolation and RT-qPCR

Total RNA was isolated from cells using the Quick-RNA MiniPrep kit (Zymo Research, Irvine, CA, USA, R1054). Reverse transcription was performed using the Takara PrimeScript RT reagent kit (Takara, Kusatsu, Japan, RR037A). A miScript SYBR Green PCR kit (Qiagen, Germany) was used to detect the expression of target genes on a Lightcycler 480 Real-Time PCR System (Roche Diagnostics GmbH, Mannheim, Germany). The relative standard curve method (2−^△△CT^) was employed to determine the relative mRNA expression, with the glyceraldehyde 3-phosphate dehydrogenase (GAPDH) gene as the reference. **Supplementary Table 2** lists the polymerase chain reaction primers used in this study.

### 2.20 Validation of protein expression levels of DRGs by immunohistochemistry

Immunohistochemistry (IHC) staining was used to detect the protein expression of DRGs in HNSCC tissues. Paraffin-embedded tissue specimens were cut into 4 μm-thick sections, deparaffinized, rehydrated with gradient ethanol, and incubated in EDTA. Endogenous peroxidase was blocked using 3% hydrogen peroxide. 10% normal goat serum was used to reduce non-specific binding. Rabbit monoclonal antibodies to DRGs (ab307587, ab235924, ab8226, ab186754; 1:1000, Abcam, UK) were used as the primary antibodies, and samples were incubated for 1 hour at room temperature. After washing with PBS, biotin-labeled secondary antibodies and streptavidin-horseradish peroxidase were added and incubated at room temperature for 10 minutes each. Samples were then stained with DAB, dehydrated, and fixed with resin.

### 2.21 Statistical analysis

The Wilcoxon rank-sum test was used to assess the expression differences of DRGs between HNSCC and adjacent tissues. Kaplan-Meier curves were analyzed using the R packages “survival” and “survminer.” Univariate and multivariate Cox regression analyses were performed using the “survival” R package. The time-dependent AUC value was calculated using the R “timeROC” package, and ROC curves were plotted using the R “survivalROC” package. Statistical significance was indicated by asterisks. A p-value < 0.05 was considered statistically significant (* p < 0.05, ** p < 0.01, *** p < 0.001). All statistical analyses were conducted using the R package.

## 3 Results

### 3.1 Identification and analysis of DRG clusters in HNSCC

**Figure 1:**
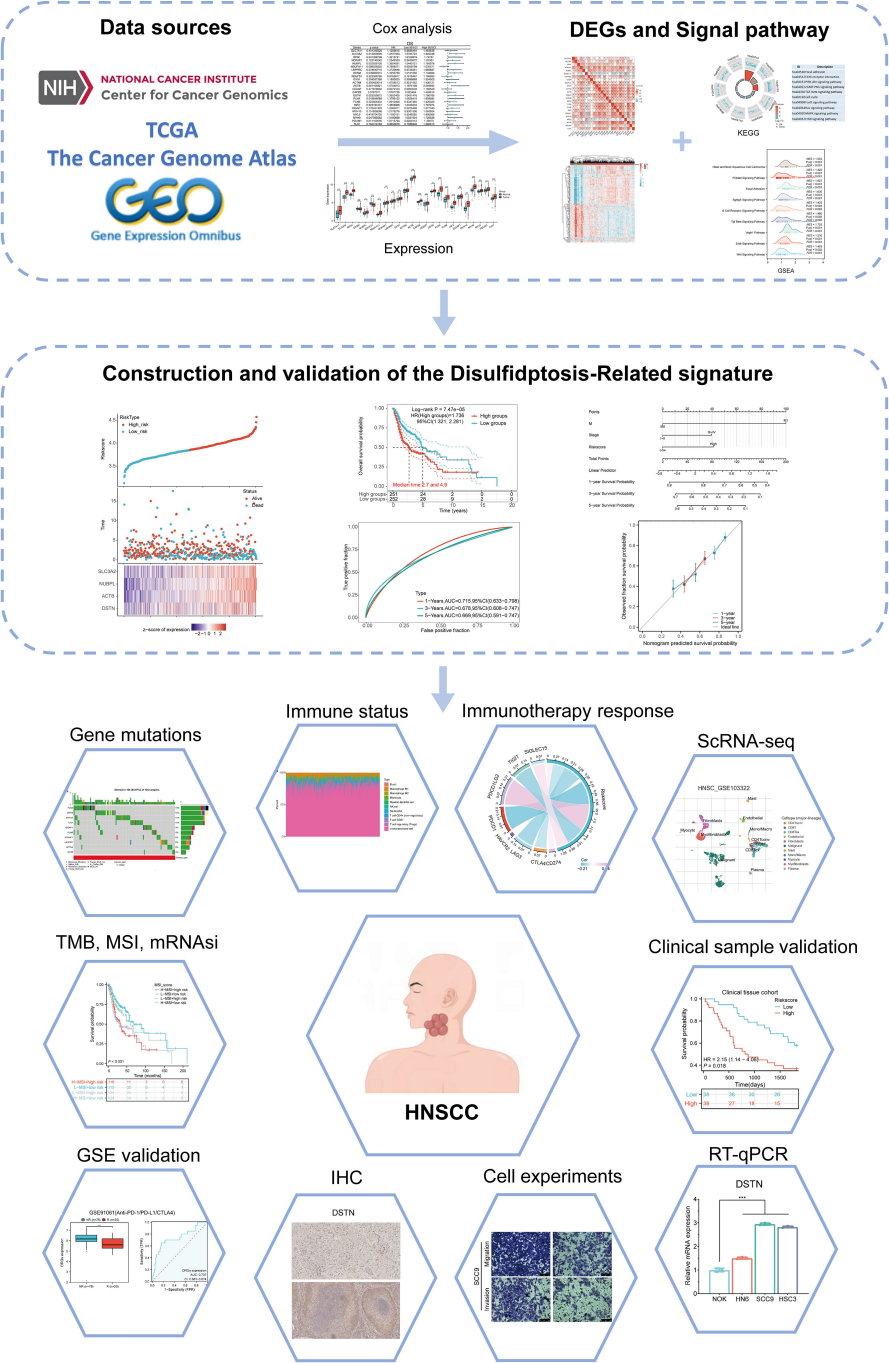
Flowchart of the present study.

**Figure 2:**
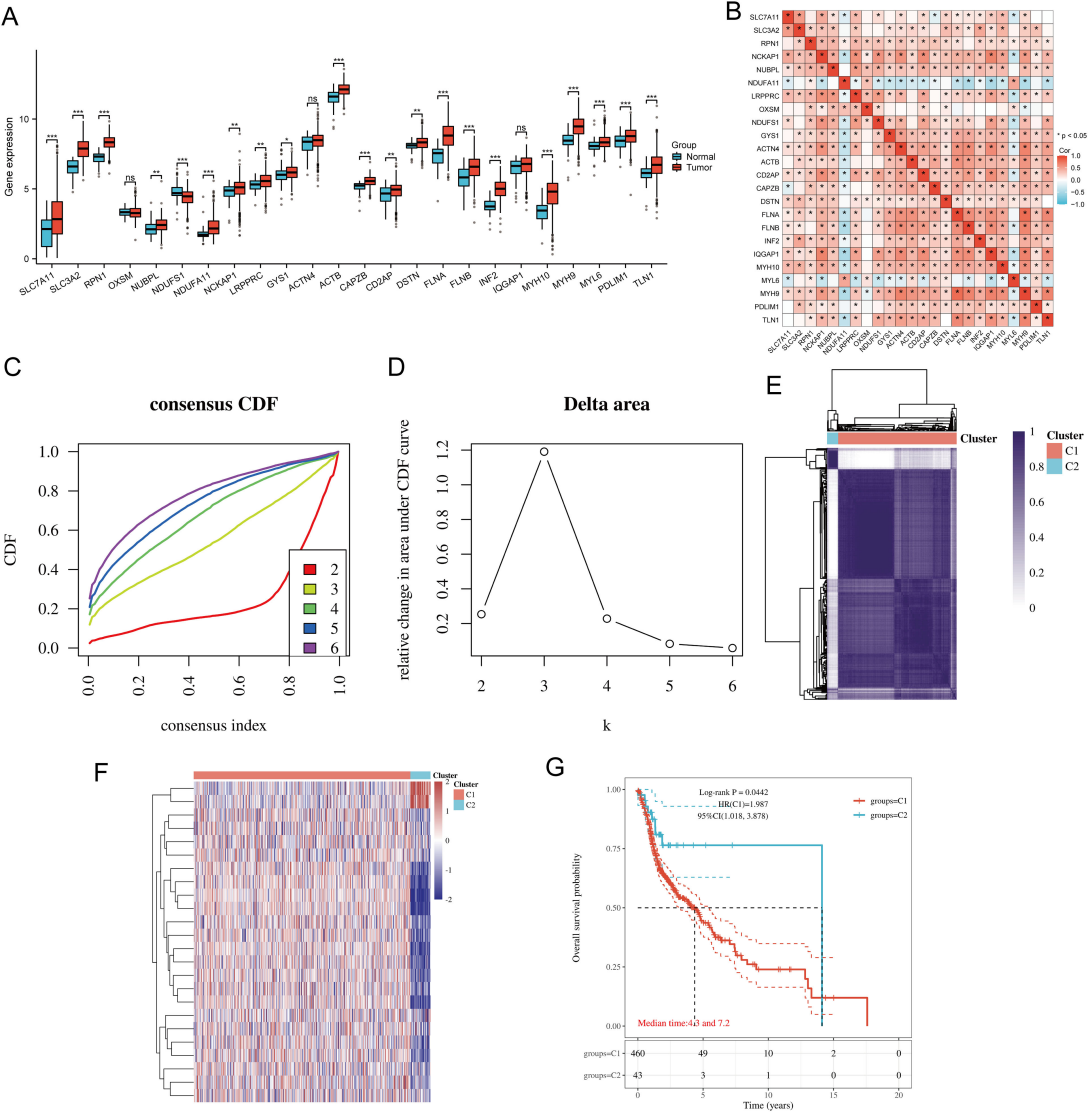
Common clusters were identified based on the expression of DRGs. **(A)** The expression levels of 24 DRGs in HNSCC and paracancerous tissues, and the quartile ranges of the upper and lower representative values of the box; the line in the box represents the median value. **(B)** Pearson’s correlation analysis for the expression of 24 DRGs in HNSCC. **(C)** Cumulative distribution function (CDF) (k = 2 - 6). **(D)** Relative change of area under CDF curve (CDF Delta area) (k = 2 - 6). **(E)** Consensus clustering matrix (k = 2). **(F)** The heat map of DRG expression in different subtypes, wherein red color represents high expression and blue color represents low expression. **(G)** Kaplan-Meier survival analysis based on two clusters. *p<0.05, **p<0.01, ***p<0.001.

The flowchart of the study is illustrated in **Figure 1**. The expression levels of 24 DRGs were compared between HNSCC tissues (n = 504) and normal tissues (n = 44) in the TCGA-HNSCC dataset. The results showed that the expression levels of SLC7A11, SLC3A2, RPN1, NUBPL, NDUFA11, NCKAP1, LRPPRC, GYS1, ACTB, CAPZB, CD2AP, DSTN, FLNA, FLNB, INF2, MYH10, MYH9, MYL6, PDLIM1, and TLN1 were upregulated in cancer tissues compared with normal tissues, whereas the expression levels of NDUFS1 were downregulated (**Figure 2A**). Additionally, most of the 24 DRGs in HNSCC samples were positively correlated (**Figure 2B**). Based on the expression levels of the 24 DRGs in HNSCC, consensus clustering was performed to classify the 504 HNSCC samples in the TCGA database. All tumor samples were divided into k (k = 2 - 6) different clusters. The cluster number was selected as two, indicating that HNSCC patients were accurately divided into two clusters (C1 and C2) (**Figures 2C–F**). The Kaplan-Meier survival curve showed that the overall survival (OS) of C2 patients was significantly worse than that of C1 patients (**Figure 2G**).

### 3.2 DEGs and functional enrichment analysis

**Figure 3:**
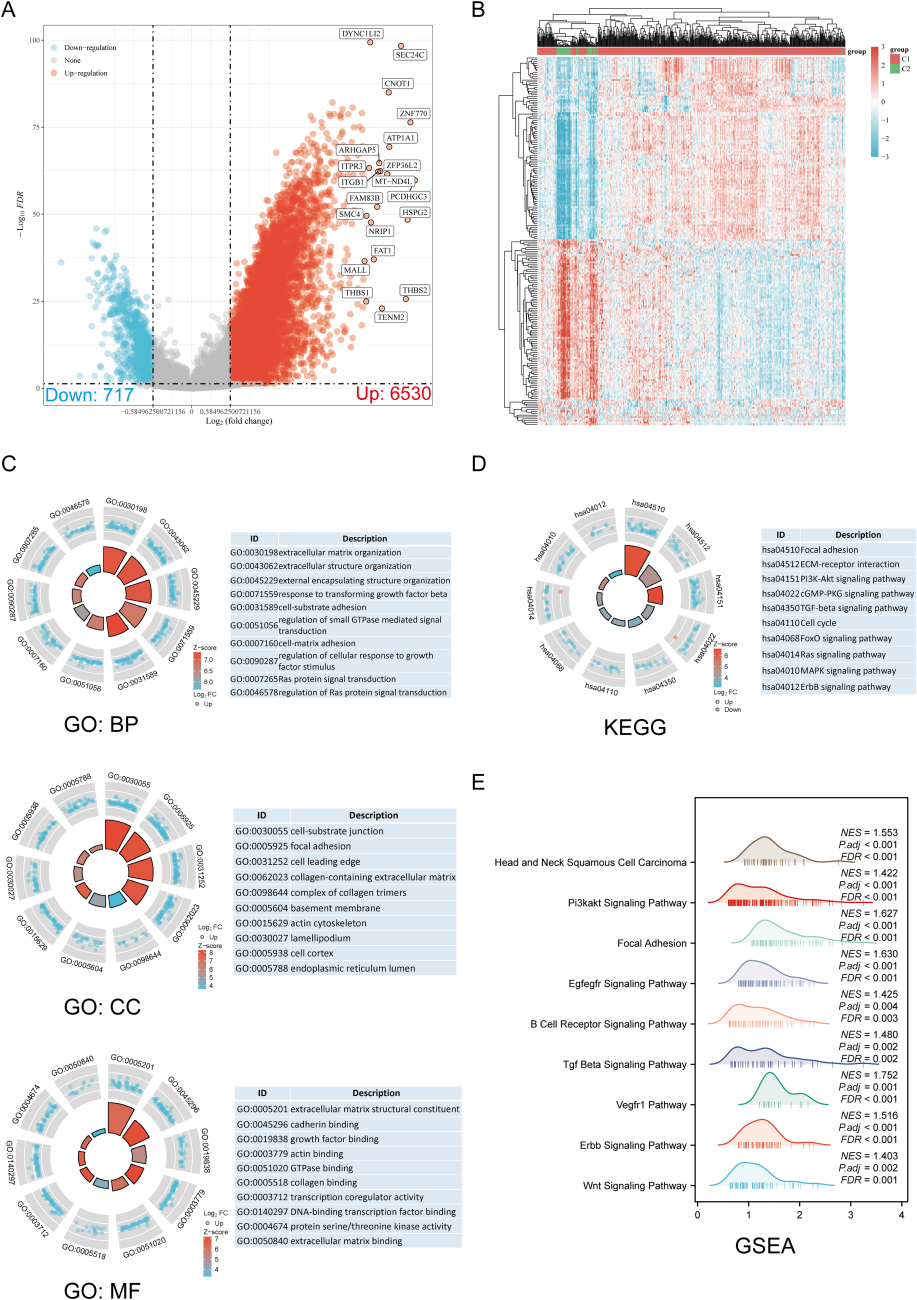
Screening of DEGs between DRG subtypes and functional enrichment analysis of DEGs. **(A)** The volcano plot of DEGs between C1 and C2 subtypes. **(B)** DEG heat map between C1 and C2 subtypes. **(C, D)** Enrichment analysis results of GO and KEGG for DEGs. **(E)** Enrichment map from GSEA.

The DEGs identified between C1 and C2 subtypes included 6530 upregulated genes and 717 downregulated genes. A volcano map (**Figure 3A**) and heat map (**Figure 3B**) were constructed for these DEGs. GO and KEGG enrichment analysis identified the upregulated and downregulated DEGs. GO analysis showed that the DEGs were mainly enriched in extracellular matrix organization, response to transforming growth factor-beta, cell-substrate adhesion, focal adhesion, collagen-containing extracellular matrix, extracellular matrix binding, collagen binding, and GTPase binding (**Figure 3C**). KEGG enrichment analysis indicated that DEGs were enriched in processes such as ECM-receptor interaction, focal adhesion, cell cycle, cGMP-PKG signaling pathway, TGF-beta signaling pathway, PI3K-Akt signaling pathway, MAPK signaling pathway, and ERBB signaling pathway (**Figure 3D**). GSEA pathway enrichment analysis showed that the expression of DRGs was closely associated with pathways including Head and Neck Squamous Cell Carcinoma, PI3K-Akt signaling pathway, focal adhesion, EGFEGFR signaling pathway, B cell receptor signaling pathway, TGF-beta signaling pathway, ERBB signaling pathway, VEGFR1 pathway, and Wnt signaling pathway (**Figure 3E**; **Supplementary Table 3**). Activation of these pathways increases the risk of tumor development and progression.

### 3.3 Correlation analysis of genetic changes

Using the GSCA database, we analyzed the percentage map of SNVs on the chart. FLNA mutation frequency was high. The oncoplot provided the SNVs of the top 10 genes among DRGs, with FLNA (18%) and MYH9 (18%) having the highest mutation frequencies, followed by MYH10 (15%), TLN1 (15%), IQGAP1 (9%), FLNB (8%), NCKAP1 (6%), LRPPRC (6%), ACTN4 (6%), and ACTB (3%) (**Supplementary Figure 1A**). Mutations were categorized, with missense mutations accounting for the largest proportion (**Supplementary Figure 1B**). Single nucleotide polymorphisms (SNPs) were more frequent than deletions (**Supplementary Figure 1C**), and C > T was the most common type of SNV (**Supplementary Figure 1D**). By calculating the number of base changes per patient, we found that the median and maximum number of mutations were 1 and 5, respectively (**Supplementary Figure 1E**). The box plot shows the number of occurrences for each variant classification (**Supplementary Figure 1F**). By considering the total number of mutations and calculating multiple hits separately, we recalculated the top 10 mutated genes (**Supplementary Figure 1G**). CNV and methylation levels are important factors that affect gene expression levels and prognosis. We analyzed the correlation between DRG CNV, methylation status, and mRNA. The results showed a significant positive correlation between DRG CNV and mRNA expression, while gene methylation levels had a negative correlation with mRNA expression (**Supplementary Figure 1H**). **Supplementary Figure 1I** shows that for some DRGs, CNV and methylation levels are significantly associated with poor prognosis in HNSCC patients. Subsequently, we analyzed the CNV landscapes of the 24 DRGs in HNSCC (**Supplementary Figure 1J**). **Supplementary Figure 1K** shows high heterozygosity deletion/amplification rates. CNV analysis revealed that DRGs had heterozygous amplification and extensive heterozygosity loss, while TLN1, RPN1, and FLNA showed high-level homozygosity amplification, and NDUFS1 and FLNB showed high-level homozygosity loss.

### 3.4 Establishing a prognostic risk model

**Figure 4:**
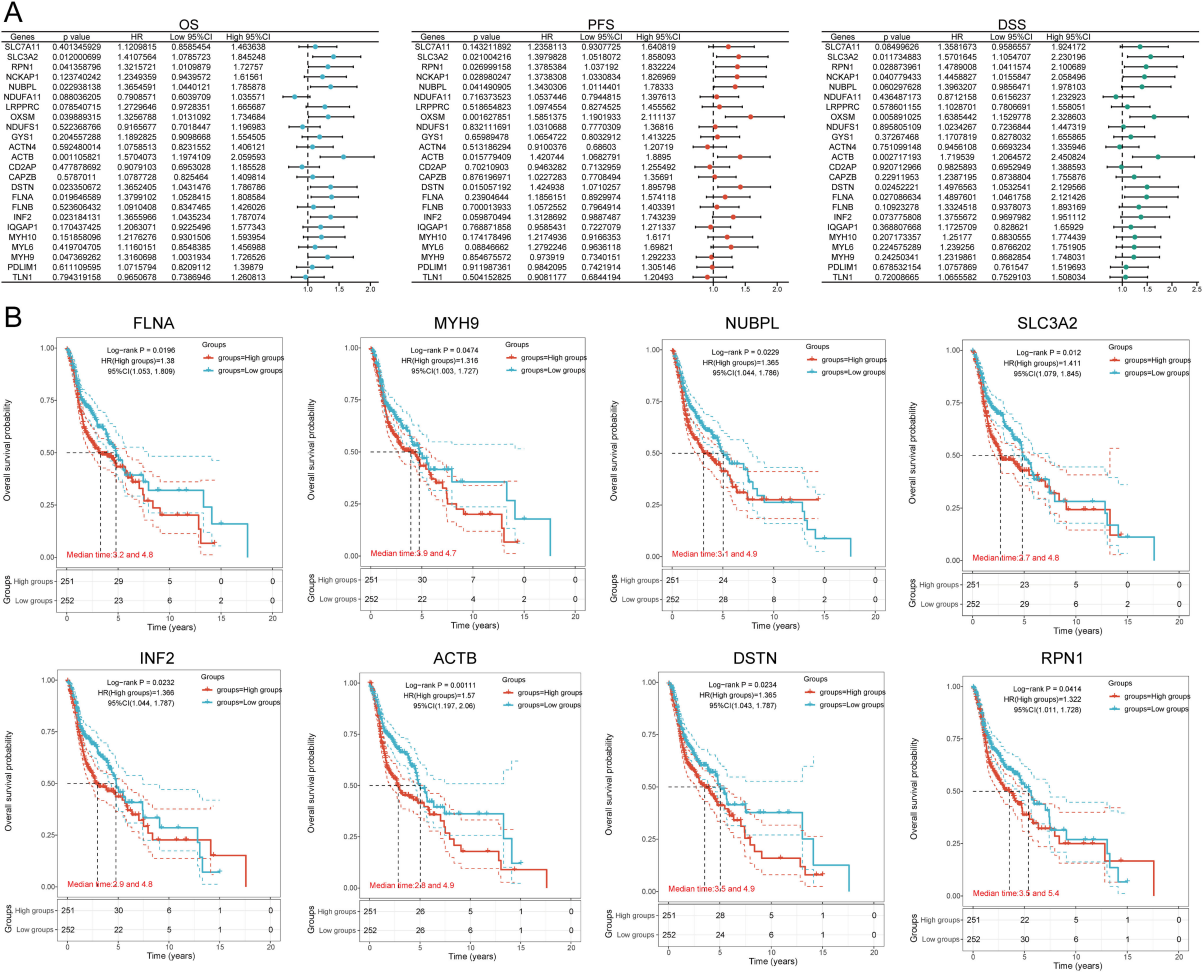
Prognostic value analysis of 24 DRGs expressions. **(A)** Univariate Cox regression analysis plots. **(B)** Prognostic value of the eight prognostic DRGs in high and low expression groups among HNSCC patients.

**Figure 5:**
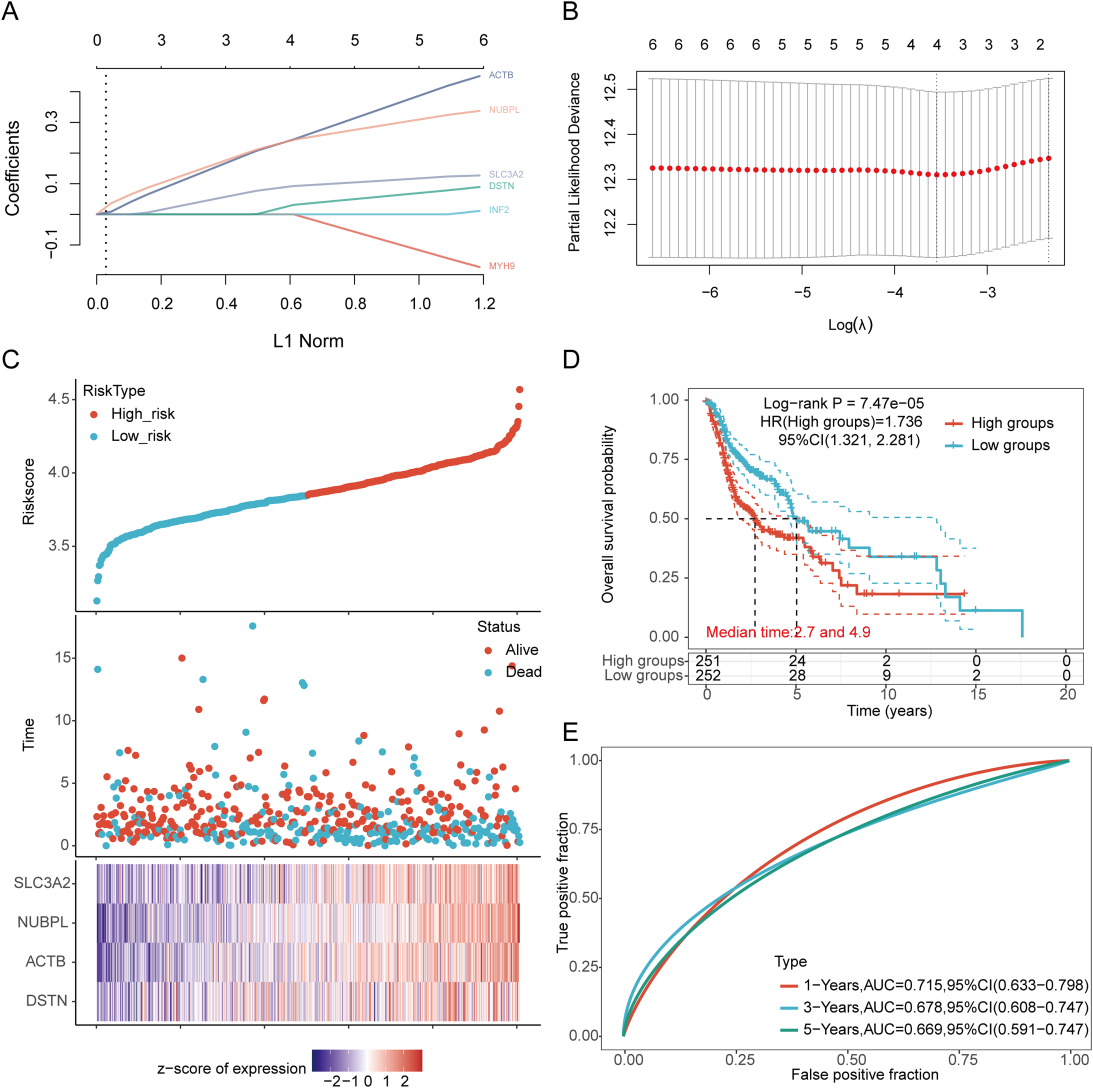
Construction of a prognostic model with the help of DRGs in HNSCC tissue. **(A)** LASSO coefficient curve of four DRGs. **(B)** Plots of the ten-fold cross-validation error rates. **(C)** Distribution of risk score, survival status, and expression of prognostic DRGs in HNSCC patients. **(D)** Overall survival curve of HNSCC patients in high/low-risk groups. **(E)** Time-dependent ROC curve for 1-, 3-, and 5-year OS for DRGs.

We identified eight genes with prognostic value (SLC3A2, RPN1, NUBPL, ACTB, DSTN, FLNA, INF2, MYH9) using univariate Cox analysis and visualized them using a forest plot, including OS, PFS, and DSS (**Figure 4A**). As shown in **Figure 4B**, the OS rate of HNSCC patients with high expression levels of SLC3A2 (HR = 1.411, p = 0.012), RPN1 (HR = 1.322, p = 0.0414), NUBPL (HR = 1.365, p = 0.0229), ACTB (HR = 1.57, p = 0.00111), DSTN (HR = 1.365, p = 0.0234), FLNA (HR = 1.38, p = 0.0196), INF2 (HR = 1.366, p = 0.0232), and MYH9 (HR = 1.316, p = 0.0474) was lower. Therefore, high expression of these genes is a prognostic factor in HNSCC patients. Based on the expression profiles of these potential prognostic biomarkers, LASSO Cox regression analysis was performed to construct an OS prognosis model based on the eight prognostic DRGs (**Figures 5A, B**). The risk score for OS in patients with HNSCC was determined as follows: Risk score = (0.0807) * SLC3A2 + (0.2193) * NUBPL + (0.2167) * ACTB + (0.0082) * DSTN. According to the risk score, TCGA-HNSCC (training cohort) patients were divided into two groups. The risk score distribution, survival status, and expression levels of the four DRGs are shown in **Figures 5C, D**. With an increase in the risk score, the risk of death increased and survival time decreased (**Figure 5C**). The Kaplan–Meier curve showed that HNSCC patients with high risk scores had lower OS rates compared with patients with low risk scores [median time = 2.7 and 4.9 years, log-rank p = 7.47e-05, HR = 1.736 (1.321, 2.281)] (**Figure 5D**). The AUCs for the 1-, 3-, and 5-year ROC curves were 0.715, 0.678, and 0.669, respectively (**Figure 5E**). The same analysis was conducted for PFS and DSS. The higher the risk score, the shorter the PFS [median time = 3 and 15 years, log-rank p = 0.000205, HR = 1.733 (1.296, 2.317)]. The AUCs for PFS predicted by 1-, 3-, and 5-year ROC curves were 0.614, 0.607, and 0.519, respectively (**Supplementary Figures 2A–C**). The DSS of patients with high expression of HNSCC was lower than that of patients with low expression [median time = 6.7 and 15 years, log-rank p = 0.000433, HR = 1.91 (1.332, 2.738)]. The AUCs for the 1-, 3-, and 5-year ROC curves were 0.613, 0.639, and 0.526, respectively (**Supplementary Figures 2D–F**). Thus, the results of the DRG-related risk scoring model showed a significant correlation with the survival rate of HNSCC patients.

### 3.5 External validation of the DRGs prognostic signature

**Figure 6:**
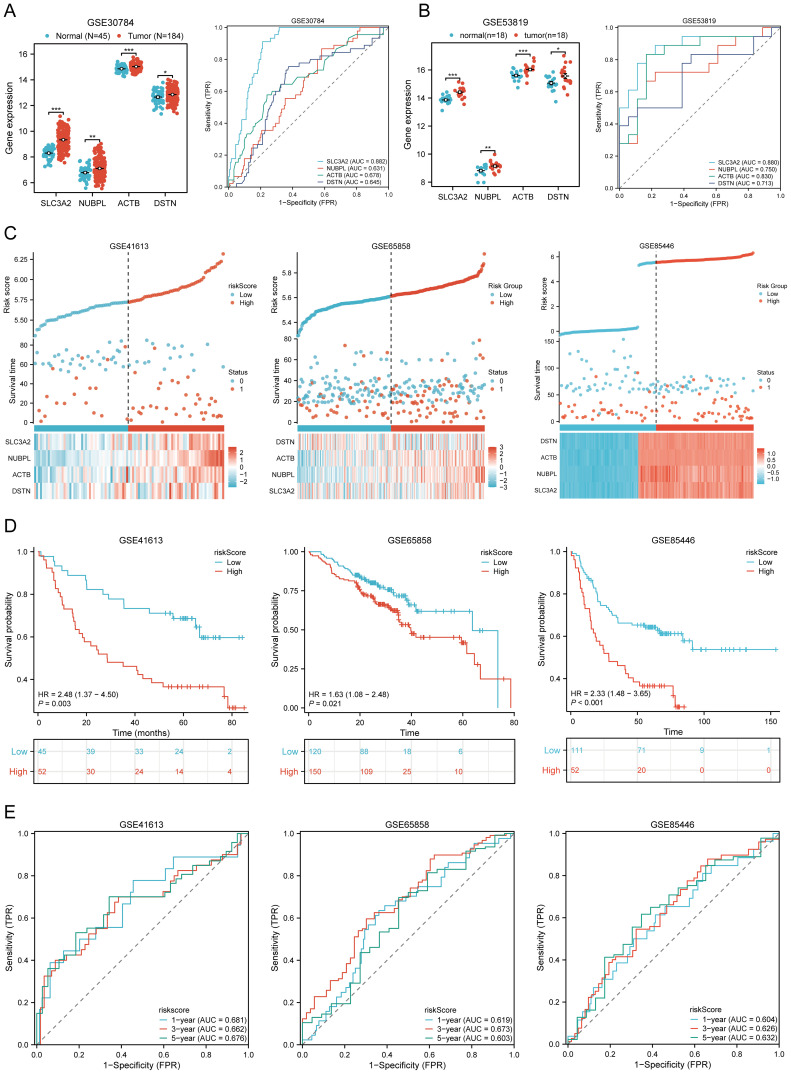
Prognostic value of DRGs signature in HNSCC patients. **(A, B)** The mRNA expression of prognostic DRGs and ROC curves to evaluate the ability of the prognostic DRGs expression to diagnose HNSCC in GSE12452 **(A)**, GSE53819 **(B)** dataset. **(C)** Distribution of risk score, survival status, and expression of prognostic DRGs for patients in low- and high-risk groups in GSE41613, GSE65858, GSE85446 dataset. **(D)** Risk score and survival probabilities in GSE41613, GSE65858, GSE85446 dataset. **(E)** Time-dependent ROC curve analyses of risk score in GSE41613, GSE65858, GSE85446 dataset. *p<0.05, **p<0.01, ***p<0.001.

We further validated the expression levels and diagnostic efficacy of prognostic DRGs using the GEO database. Compared with the low expression group, the expression levels of prognostic DRGs in the high expression group were significantly upregulated in the GSE30784 and GSE53819 datasets. In dataset GSE30784, the AUC values of SLC3A2, NUBPL, ACTB, and DSTN were 0.882, 0.631, 0.678, and 0.645, respectively (**Figure 6A**). In dataset GSE53819, the AUC values of SLC3A2, NUBPL, ACTB, and DSTN were 0.880, 0.750, 0.830, and 0.713, respectively (**Figure 6B**). The four prognostic DRGs (SLC3A2, NUBPL, ACTB, and DSTN) consistently showed good sensitivity and specificity in diagnosing HNSCC. To verify the predictive value of the four-gene signature, the GSE41613, GSE65858, and GSE85446 datasets were used as external validation cohorts. We calculated the risk scores for each patient using the same formula, consistent with the results of the TCGA cohort. The distribution of risk scores, survival time, and DRG expression in each HNSCC patient is shown in **Figure 6C**. In the validation set, OS was significantly worse in patients with the high-risk group compared to those with the low-risk group (p = 0.003, p = 0.021, p < 0.001) (**Figure 6D**). The AUCs for 1-year, 3-year, and 5-year OS were 0.681, 0.662, and 0.676 in the GSE41613 dataset, respectively. The AUCs for 1-year, 3-year, and 5-year OS were 0.604, 0.626, and 0.632 in the GSE85446 dataset, respectively. The AUCs for 1-year, 3-year, and 5-year OS were 0.619, 0.673, and 0.603 in the GSE65858 dataset, respectively (**Figure 6E**). To sum up, these results confirm the effectiveness of our risk scoring model. The four-gene signature can predict survival rates in HNSCC. Taken together, these results confirm the validity of our risk score model, and that the DRGs prognostic signature can predict OS in HNSCC.

### 3.6 Clinical correlation analysis

Based on the above-mentioned prognostic signature, we explored the survival analysis of clinical pathological features between high-risk and low-risk groups (**Supplementary Table 4**). Subgroup survival analysis showed that the high-risk group significantly affected the overall survival time of patients who were Age > 60 (p < 0.001, HR = 2.17 (1.50 − 3.14)), Female (p < 0.001, HR = 2.58 (1.52 − 4.37)), Male (p = 0.013, HR = 1.51 (1.09 − 2.10)), White (p < 0.001, HR = 1.74 (1.30 − 2.34)), Grade 1-2 (p = 0.008, HR = 1.55 (1.12 − 2.14)), Grade 3-4 (p = 0.010, HR = 2.10 (1.20 − 3.67)), Stage III-IV (p < 0.001, HR = 1.80 (1.33 − 2.43)), M0 Status (p < 0.001, HR = 1.78 (1.34 − 2.35)), N0 Status (p = 0.020, HR = 1.61 (1.08 − 2.40)), N1-3 Status (p < 0.001, HR = 2.07 (1.40 − 3.04)), T3-4 Status (p < 0.001, HR = 1.87 (1.32 − 2.63)), Neoadjuvant N0 (p < 0.001, HR = 1.69 (1.28 − 2.23)), Smoking Yes (p < 0.001, HR = 1.87 (1.32 − 2.63)) (**Supplementary Figures 3A–M**). However, factors such as Age <= 60 (p = 0.107, HR = 1.41 (0.93 − 2.14)), Asian + Black (p = 0.484, HR = 0.75 (0.33 − 1.69)), Stage I-II (p = 0.246, HR = 1.50 (0.76 − 2.95)), Neoadjuvant Yes (p = 0.152, HR = 4.81 (0.56 − 41.07)), Radiation N0 (p = 0.870, HR = 0.93 (0.39 − 2.20)), Radiation Yes (p = 0.061, HR = 1.77 (0.97 − 3.23)), Smoking N0 (p = 0.184, HR = 1.51 (0.82 − 2.75)), and T1-2 Status (p = 0.104, HR = 1.48 (0.92 − 2.39)) were not significantly associated with the overall survival time of HNSCC patients (**Supplementary Figures 4A–H**). This suggests that these factors play an important role in determining the survival outcomes of patients with HNSCC and should be considered when developing treatment strategies.

### 3.7 Establishment and validation of a predictive nomogram

**Figure 7:**
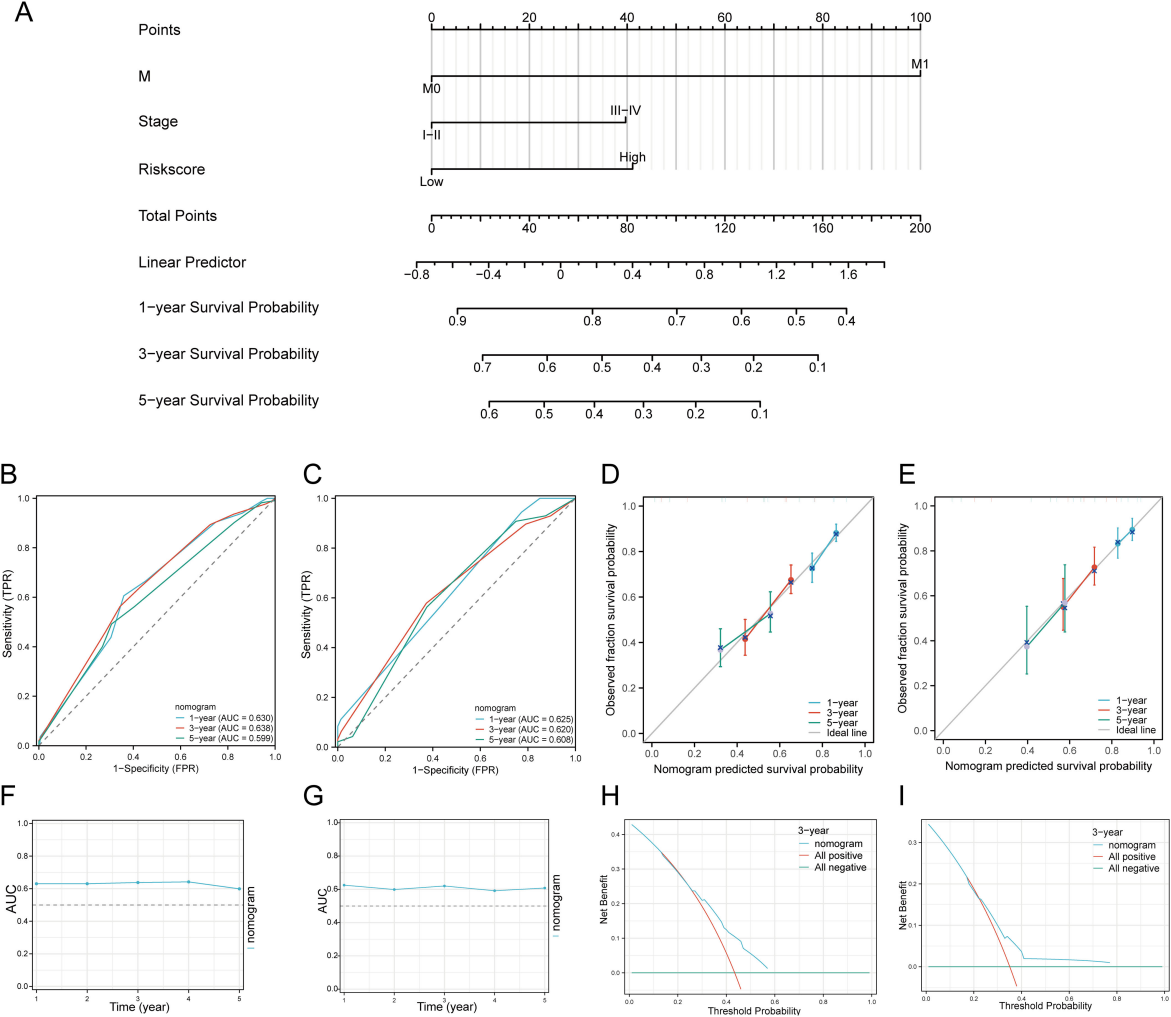
Construction of a predictive nomogram. **(A)** Nomogram for predicting 1-, 3-, and 5-year OS of HNSCC patients. **(B, C)** ROC curves for predicting 1-, 3-, and 5-year OS in the TCGA and GEO datasets. **(D, E)** Calibration curve of OS nomogram model in the discovery group in the TCGA and GEO datasets. **(F, G)** Time-dependent AUC curve shows the nomogram to predict OS performance in the TCGA and GEO datasets. (The diagonal dotted line represents the ideal nomogram). **(H, I)** DCA curves for the nomogram in the TCGA and GEO datasets.

We first performed univariate and multivariate Cox analyses to establish a predictive nomogram that integrates the DRGs risk score with other prognosis-related clinical factors. In univariate Cox regression analysis, M status (HR = 4.819, 95% CI = 1.775 - 13.083, p = 0.002), Stage (HR = 0.568, 95% CI = 0.394 - 0.821, p = 0.003), and risk score (HR = 0.576, 95% CI = 0.438 - 0.757, p < 0.001) were associated with OS in HNSCC patients. In multivariate Cox regression analysis, M status (HR = 3.919, 95% CI = 1.414 - 10.861, p = 0.009), Stage (HR = 0.560, 95% CI = 0.373 - 0.841, p = 0.005), and risk score (HR = 0.534, 95% CI = 0.403 - 0.709, p < 0.001) were shown to be independent predictors of OS in HNSCC patients (**Supplementary Table 5**). The risk score, M status, and Stage were then integrated to construct a nomogram for predicting 1-, 3-, and 5-year OS in HNSCC patients. The results of the predictive nomogram showed that 1-, 3-, and 5-year OS [C-index: 0.613 (0.594-0.633)] (**Figure 7A**), PFS [C-index: 0.603 (0.583-0.623)] (**Supplementary Figures 5A–E**), and DSS [C-index: 0.645 (0.622-0.669)] (**Supplementary Figures 6A–E**). The AUC values for 1-, 3-, and 5-year ROC curves were 0.630, 0.638, and 0.599, respectively (**Figure 7B**). Calibration curves showed good consistency between predicted and observed values, especially for 3-year OS (**Figure 7D**) and time-dependent AUC curves (**Figure 7F**). Finally, we performed DCA curves to assess the clinical utility of the nomogram, indicating its value in predicting survival rates (**Figure 7H**). In the GEO validation cohort, the AUC values for 1-, 3-, and 5-year OS were 0.625, 0.620, and 0.608, respectively (**Figure 7C**). Calibration curves and the time-dependent AUC for the nomogram model also maintained good performance in predicting patient OS (**Figures 7E, G**). DCA showed that the nomogram also provided clinical net benefits (**Figure 7I**). Thus, in both the TCGA cohort and GEO external validation cohort, the nomogram incorporating DRG risk score and clinical characteristics (M status and Stage) appears to accurately predict short-term and long-term OS in HNSCC patients. Overall, these results indicate that the constructed nomogram has predictive accuracy for the prognosis of HNSCC patients and may bring significant clinical benefits.

### 3.8 Association of tumor immune cell infiltration with the disulfidptosis-related prognostic signature in HNSCC

**Figure 8:**
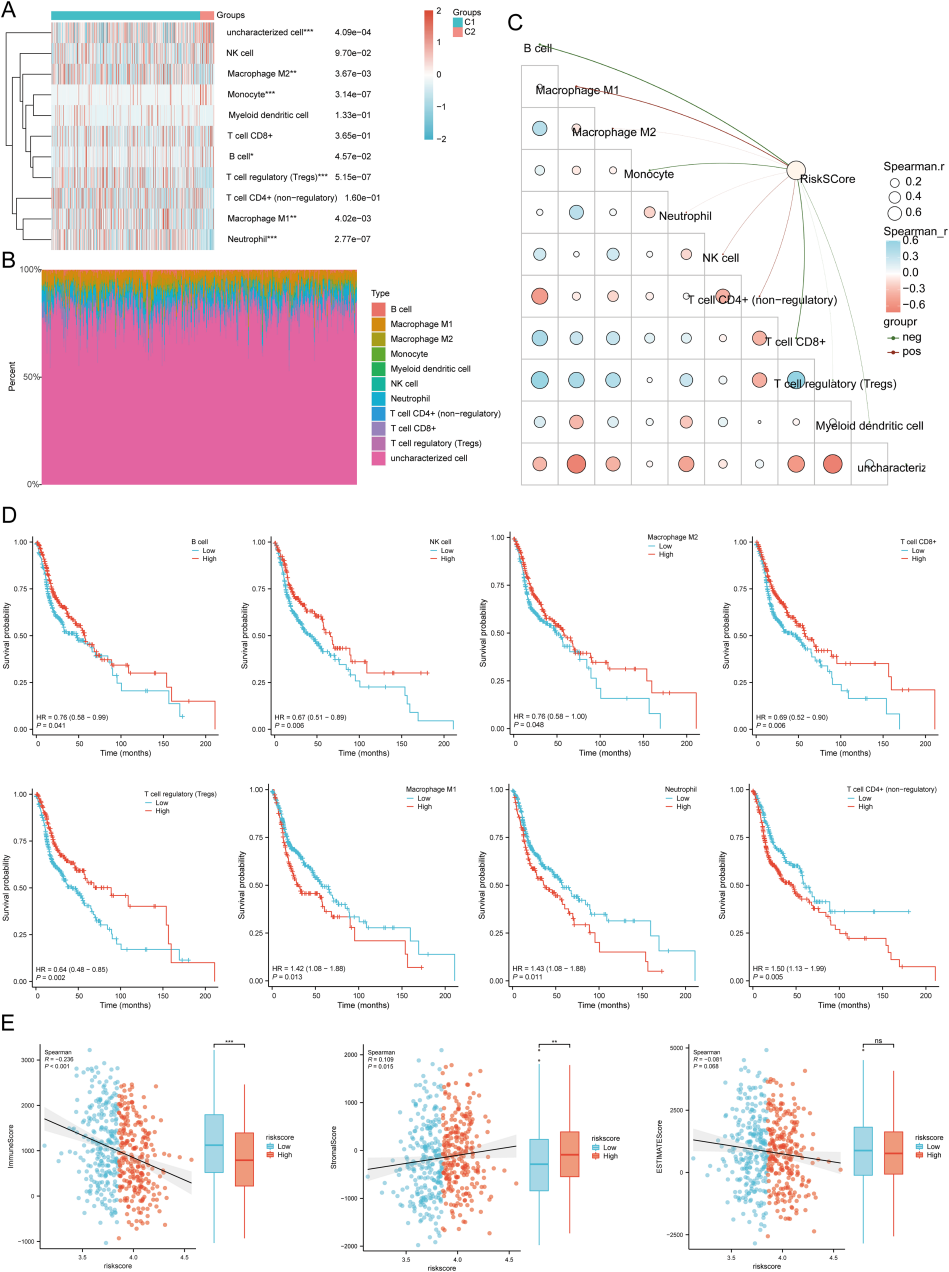
Relationship between the expression level of DRGs and immune infiltration in the tumor microenvironment. **(A, B)** Comparison of immune scores between C1 and C2 subtypes in TCGA (QUANTISEQ); the abscissa represents the type of immune cell infiltration, and the ordinate represents the distribution of the immune infiltration score in different groups. **(C)** The correlation analysis between Riskscore and immunoscore (QUANTISEQ). **(D)** The relationship between the level of immune cell infiltration and survival rate, including B cells, NK cells, macrophages M2, T cell CD8+, T cell regulatory (Tregs), Macrophage M1, Neutrophil, T cell CD4+ (non-regulatory). **(E)** Correlation between Riskscore and three ESTIMATE, and Differences in ESTIMATE between the high and low expression groups of the four prognostic DRGs in HNSCC. *p<0.05, **p<0.01, ***p<0.001.

We used six algorithms to observe the differences in immune cells between C1 and C2 subtypes of HNSCC samples. The QUANTISEQ algorithm showed significant differences in Macrophage M2 (P = 0.004), Monocyte (P = 3.14E-07), Macrophage M1 (P = 0.004), B cell (P = 0.0457), T cell regulatory (Tregs) (P = 5.15E-07), Neutrophil (P = 2.77E-07), and uncharacterized cell (P = 0.0004) between the two subtypes (**Figures 8A, B**). Further analysis using the QUANTISEQ algorithm found significant associations between risk scores and various immune cell populations. Risk scores were negatively correlated with B cells (P = 4.44E-11, Cor = -0.2880), Monocytes (P = 7.49E-08, Cor = -0.2368), T cells CD8+ (P = 2.63E-09, Cor = -0.2612), uncharacterized cells (P = 0.0155, Cor = -0.1079), and Myeloid dendritic cells (P = 0.0011, Cor = -0.1455), and positively correlated with Macrophage M1 (P = 8.39E-10, Cor = 0.2700), NK cells (P = 0.0048, Cor = 0.1253), and T cells CD4+ (non-regulatory) (P = 0.0003, Cor = 0.1616) (**Figure 8C**). Similarly, significant differences in the distribution of immunologic infiltration scores between C1 and C2 subtypes were also observed using the TIMER, xCell, MCP-counter, CIBERSORT, and EPIC algorithms (**Supplementary Figures 7A–E**). There was also a correlation between risk scores and various immune cell populations (**Supplementary Figures 8A–E**). It has been reported that immune infiltration may affect patient prognosis. Therefore, we conducted a survival analysis of the different types of immune cells mentioned above and found that high infiltration levels of B cells, NK cells, Macrophage M2, T cells CD8+, and Tregs were associated with good prognosis, while high infiltration levels of Macrophage M1, Neutrophils, and T cells CD4+ (non-regulatory) were associated with lower OS rates (**Figure 8D**). Considering the differences in immune cell infiltration, we further analyzed the correlation between the risk score model and three ESTIMATE scores. The analysis showed a significant negative correlation between the risk score and ImmuneScore (P < 0.001, Cor = -0.236), and a positive correlation with StromalScore (P = 0.015, Cor = 0.109), but no significant correlation with ESTIMATE scores (P = 0.068, Cor = -0.081) (**Figure 8E**).

Using the ssGSEA method, immune cell infiltration between high and low expression groups of SLC3A2, NUBPL, ACTB, and DSTN was analyzed (**Supplementary Figure 9A**). In addition, in the low-risk score group, the expression levels of aDC, B cells, CD8 T cells, Cytotoxic cells, DC, Mast cells, NK CD56dim cells, pDC, T cells, TFH, and Th17 cells were higher than those in the high-risk score group. However, Eosinophils, Macrophages, Neutrophils, NK cells, Tcm, Tgd, Th1 cells, and Th2 cells were expressed at higher levels in the high-risk score group, with statistical differences (**Supplementary Figure 9B**). Correlation analysis showed that SLC3A2 expression was positively correlated with Tgd and negatively correlated with Cytotoxic cells, T cells, B cells, and CD8 T cells; NUBPL expression was positively correlated with T helper cells, NK cells, Tcm, and Th2 cells, and negatively correlated with Cytotoxic cells, PDC, NK CD56dim cells, and T cells; ACTB expression was positively correlated with Macrophages, Tgd, Th1 cells, Neutrophils, and Th2 cells, and negatively correlated with B cells, NK CD56bright cells, PDC, and CD8 T cells; DSTN expression was positively correlated with Tgd and negatively correlated with Cytotoxic cells, T cells, B cells, and NK CD56dim cells (**Supplementary Figure 9C**). In addtion, TIMER database analysis showed that SLC3A2 was positively correlated with B cells, CD4+ T cells, neutrophils, macrophages and dendritic cells. NUBPL was positively correlated with tumor purity, neutrophils. ACTB was positively correlated with tumor purity, B cells, CD4+ T cells, CD8+ T cells, neutrophils, macrophages and dendritic cells. DSTN was also positively correlated with B cells, CD4+ T cells, CD8+ T cells, neutrophils, macrophages and dendritic cells in HNSCC (**Supplementary Figure 9D**). These results showed a significant correlation between DRGs and tumor immune infiltration, indicating potential targets for immunotherapy.

### 3.9 Immunotherapy response analysis

**Figure 9:**
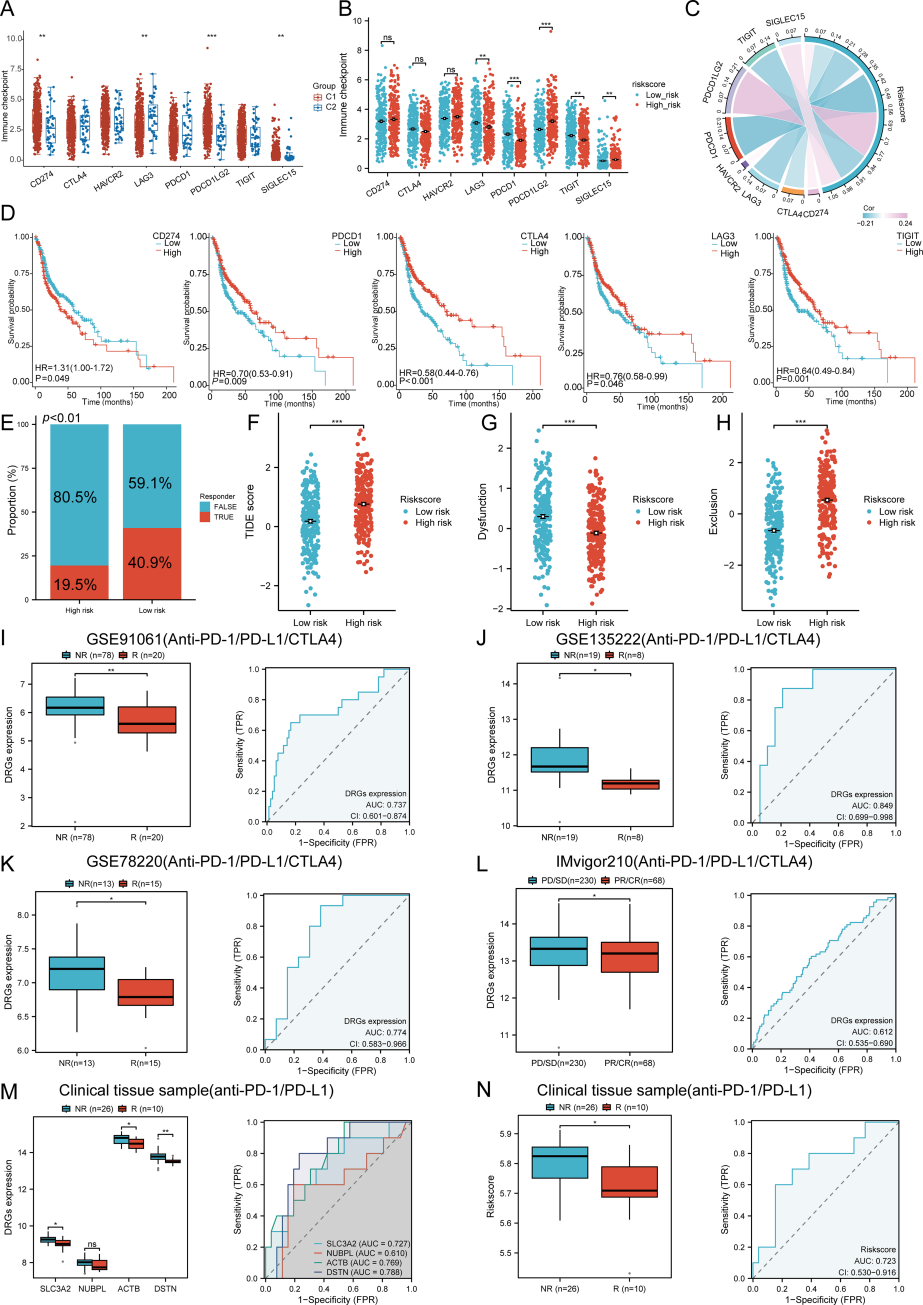
The correlation between the expression of the prognostic DRGs and immunogenicity. **(A)** The expression distributions of eight immune checkpoint-related genes in HNSCC subtypes. **(B)** Differences in immune checkpoint-related genes between high and low riskscore group. **(C)** Correlation between the prognostic DRGs in HNSCC and immune checkpoint-related genes. **(D)** The survival analysis of immune checkpoint-related genes. **(E)** The prediction of response rates of immunotherapies in patients with DRGs high and low riskscore. **(F)** Different reactions of DRGs high and low riskscore groups to immune checkpoint blocking in TIDE score. **(G)** Differences of DRGs high and low riskscore groups in TIDE Dysfunction score. **(H)** Differences of DRGs high and low riskscore groups in TIDE Exclusion score. **(I–L)** Prediction of immune response and ROC analysis of DRGs riskscore for prediction of ICI responsiveness in GSE91061, GSE135222, GSE78220, IMvigor210 dataset. **(M)** Four prognostic DRGs expression differences between patients with NR and R in clinical tissue cohort, respectively; ROC analysis of four prognostic DRGs for prediction of ICI responsiveness in clinical tissue cohort, respectively; **(N)** Riskscore differences between patients with NR and R in clinical tissue cohort; ROC analysis of riskscore for prediction of ICI responsiveness in clinical tissue cohort. (NR: not responding to immunotherapy. R: respond to immunotherapy). n.s. no significance (p > 0.05), *p<0.05, **p<0.01, ***p<0.001.

We analyzed the differences in expression between the two subtypes based on eight immune checkpoint-related genes. The results showed significant differences in the expression levels of CD274 (P < 0.01), LAG3 (p < 0.01), PDCD1LG2 (p < 0.001), and SIGLEC15 (p < 0.01) between the two subtypes. In group C1, the expression levels of CD274, PDCD1LG2, and SIGLEC15 were higher than those in group C2, with statistical significance (**Figure 9A**). We then explored the expression distribution of immune checkpoint-related genes in high and low risk score groups. The results showed significant differences in LAG3, PDCD1, PDCD1LG2, TIGIT, and SIGLEC15 between the high and low risk score groups (**Figure 9B**). Further analysis of the relationship between the expression of prognostic DRGs and immune checkpoint members in the TCGA database showed that the risk score was positively correlated with PDCD1LG2 (P = 7.6256E-08, Cor = 0.2369), SIGLEC15 (P = 0.0140, Cor = 0.1095), and CTLA4 (P = 0.0152, Cor = -0.1081). It was negatively correlated with LAG3 (P = 0.0002, Cor = -0.1659), PDCD1 (P = 1.1598E-06, Cor = -0.2148), and TIGIT (P = 0.0001, Cor = -0.1699) (**Figure 9C**). Survival analysis of immune checkpoint members showed that high levels of CTLA4 (p < 0.001, HR = 0.58 (0.44 - 0.76)), PDCD1 (p = 0.009, HR = 0.70 (0.53 - 0.91)), TIGIT (p = 0.001, HR = 0.64 (0.49 - 0.84)), and LAG3 (p = 0.046, HR = 0.76 (0.58 - 0.99)) were associated with good prognosis, while CD274 (p = 0.049, HR = 1.31 (1.00 - 1.72)) was associated with lower OS rates (**Figure 9D**). Additionally, we used the TIDE database and GSE91061, GSE135222, GSE78220, IMvigor210 datasets to predict the response of DRGs to immunotherapy. The results showed that the prediction of response rates to immunotherapies in patients with low risk scores was higher than that in the high risk group (p < 0.05) (**Figure 9E**). The low risk score group responded better to immune checkpoint blocking than the high risk score group (**Figure 9F**). TIDE Dysfunction scores were elevated in the low group (**Figure 9G**), and TIDE Exclusion scores were lower in the low group (**Figure 9H**). In the GSE91061, GSE135222, GSE78220, and IMvigor210 datasets, the AUC results further confirmed the accuracy of DRG expression in predicting immune response, with AUC values of 0.737, 0.849, 0.774, and 0.612, respectively (**Figures 9I–L**). To confirm the predictive role of DRGs risk score in immune therapy response in clinical tissue samples of HNSCC, 36 advanced HNSCC patients receiving anti-PD-1/PD-L1 therapy were analyzed. The results indicated that the expression of the four prognostic DRGs was lower in patients who achieved complete or partial remission (CR/PR). The AUC values of SLC3A2, NUBPL, ACTB, and DSTN were 0.727, 0.610, 0.769, and 0.788, respectively (**Figure 9M**). The low-risk group based on the prognostic model had a higher proportion of patients in the CR/PR group, with an AUC value of 0.723 for the risk score (**Figure 9N**). Therefore, DRGs risk score has significant potential in predicting immune therapy response, suggesting that patients with a low DRGs risk score may be more sensitive to ICI treatment. Overall, these results imply that DRGs low-risk score groups are more likely to have an immune response and respond to immunotherapy.

### 3.10 TMB, MSI, mRNAsi, and drug sensitivity analysis

**Figure 10:**
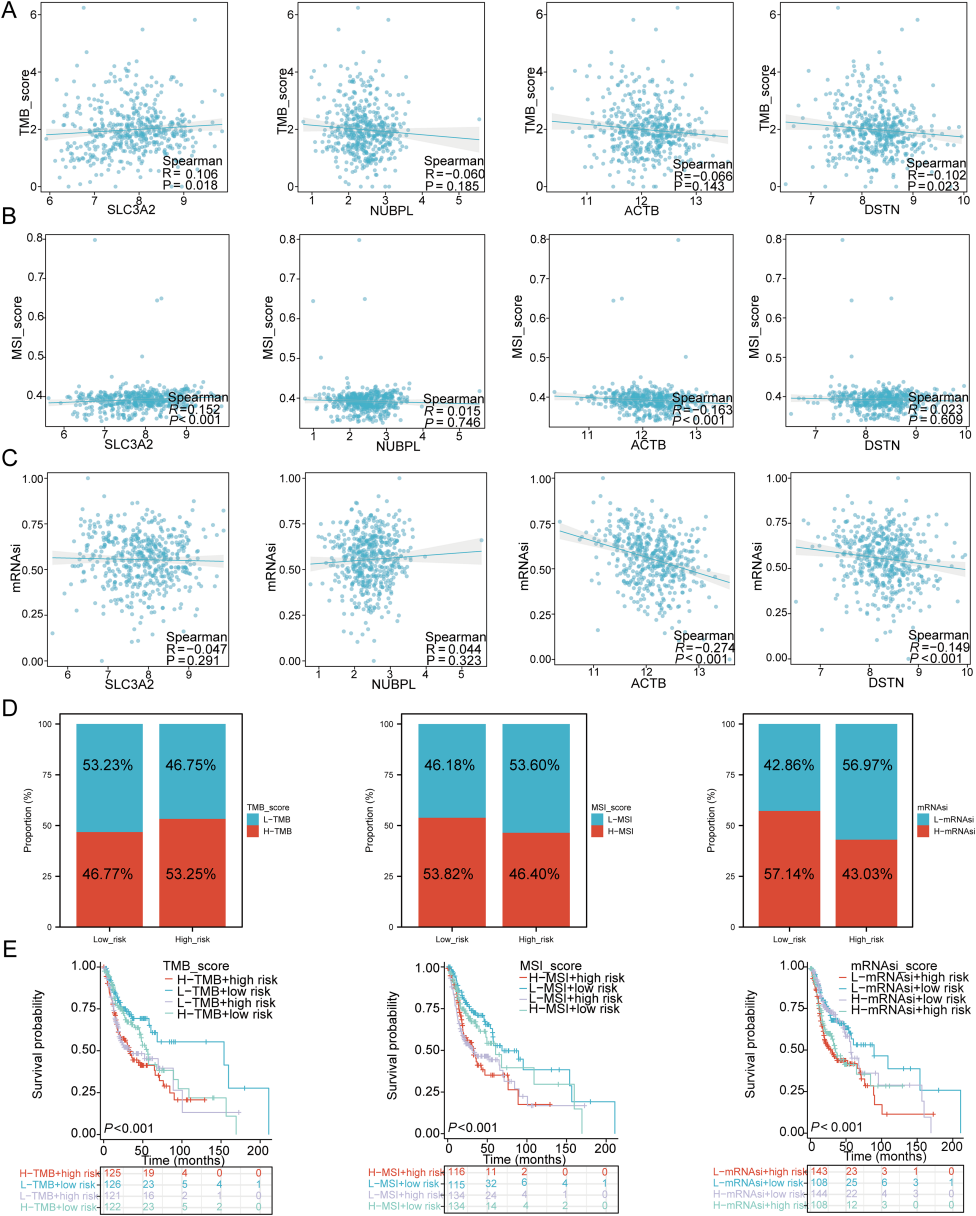
TMB, MSI, mRNAsi, and ESTIMATE analysis. **(A)** Correlation between the expression of four prognostic DRGs and TMB in HNSCC. **(B)** Correlation between the expression of four prognostic DRGs and MSI in HNSCC. **(C)** Correlation between the expression of four prognostic DRGs and mRNAsi in HNSCC. **(D)** Distribution of TMB, MSI, and mRNAsi in high-risk and low-risk groups. **(E)** Kaplan-Meier curves of four groups classified by risk score and TMB, MSI, mRNAsi in HNSCC.

To investigate the role of DRGs in immune mechanisms and responses within the TME, we assessed the correlation between the risk score model and TMB, MSI, and mRNAsi. The results showed that SLC3A2 (P = 0.018, Cor = 0.106) was positively correlated with TMB, while DSTN (P = 0.023, Cor = -0.102) was negatively correlated with TMB (**Figure 10A**). SLC3A2 (P < 0.001, Cor = 0.152) was positively correlated with MSI, while ACTB (P < 0.001, Cor = -0.613) was negatively correlated with MSI (**Figure 10B**). The expression levels of ACTB (P < 0.001, Cor = -0.274) and DSTN (P < 0.001, Cor = -0.149) were negatively correlated with mRNAsi (**Figure 10C**). Next, we analyzed the distribution of TMB, MSI, and mRNAsi in the high-risk and low-risk groups of HNSCC patients. The results revealed that the proportion of patients with high TMB was higher in the high-risk group compared to the low-risk group, while MSI and mRNAsi were more prevalent in the low-risk group (**Figure 10D**). We further performed survival analysis combining risk scores with TMB, MSI, and mRNAsi, dividing patients into four subgroups for survival assessment. The overall survival (OS) was better in the low TMB + low-risk score group compared to the high TMB + high-risk score group (P < 0.001). Similarly, patients in the high MSI + high-risk group had a worse prognosis compared to those in the low MSI + low-risk group (P < 0.001), and the OS of patients in the low mRNAsi + low-risk group was better than that of those in the high mRNAsi + high-risk group (P < 0.001) (**Figure 10E**).

**Figure 11:**
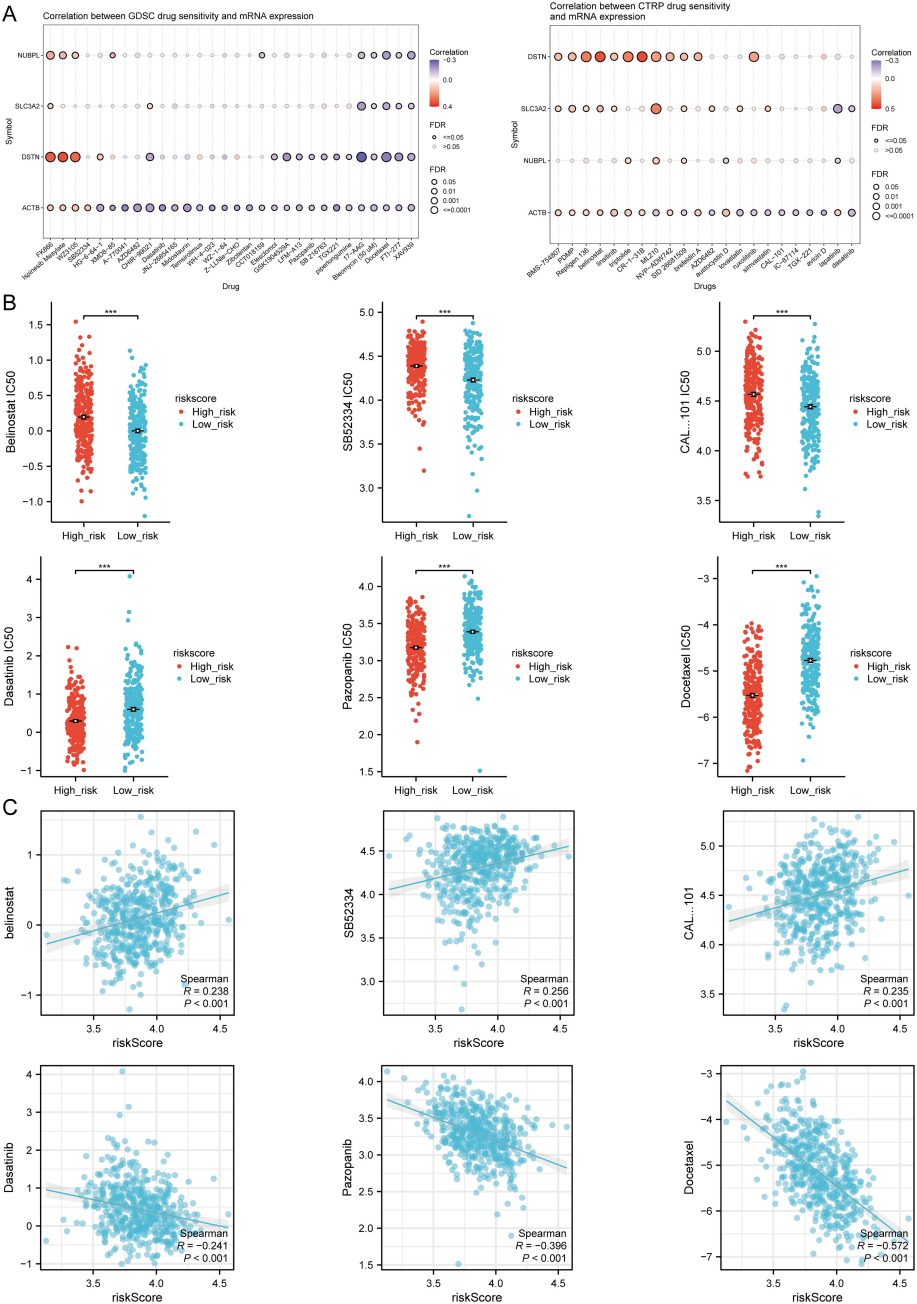
Drug sensitivity analysis. **(A)** Predictive antitumor drugs based on the three prognostic DRGs expression in HNSCC from the GDSC and CTRP datasets. **(B)** The distribution of IC50 scores in the high and low risk groups. **(C)** Spearson correlation analysis of IC50 score and riskscore. ***p<0.001.

Finally, to fully explore the potential value of new therapeutic targets for SLC3A2, NUBPL, ACTB, and DSTN, we selected some drugs from the GDSC and CTRP databases that showed a significant correlation between the risk score model and drug sensitivity (**Figure 11A**). In high-risk HNSCC, the sensitivity of belinostat, SB52334, and CAL-101 was significantly higher than in the low-risk group, while Dasatinib, Pazopanib, and Docetaxel showed higher sensitivity in low-risk HNSCC (**Figure 11B**). The results of Spearman correlation analysis showed that the expression levels of the risk score were positively correlated with belinostat, SB52334, and CAL-101, but negatively correlated with Dasatinib, Pazopanib, and Docetaxel (**Figure 11C**). Therefore, the drugs mentioned above may be potential therapeutic options for HNSCC.

### 3.11 Single cell RNA data analysis

**Figure 12:**
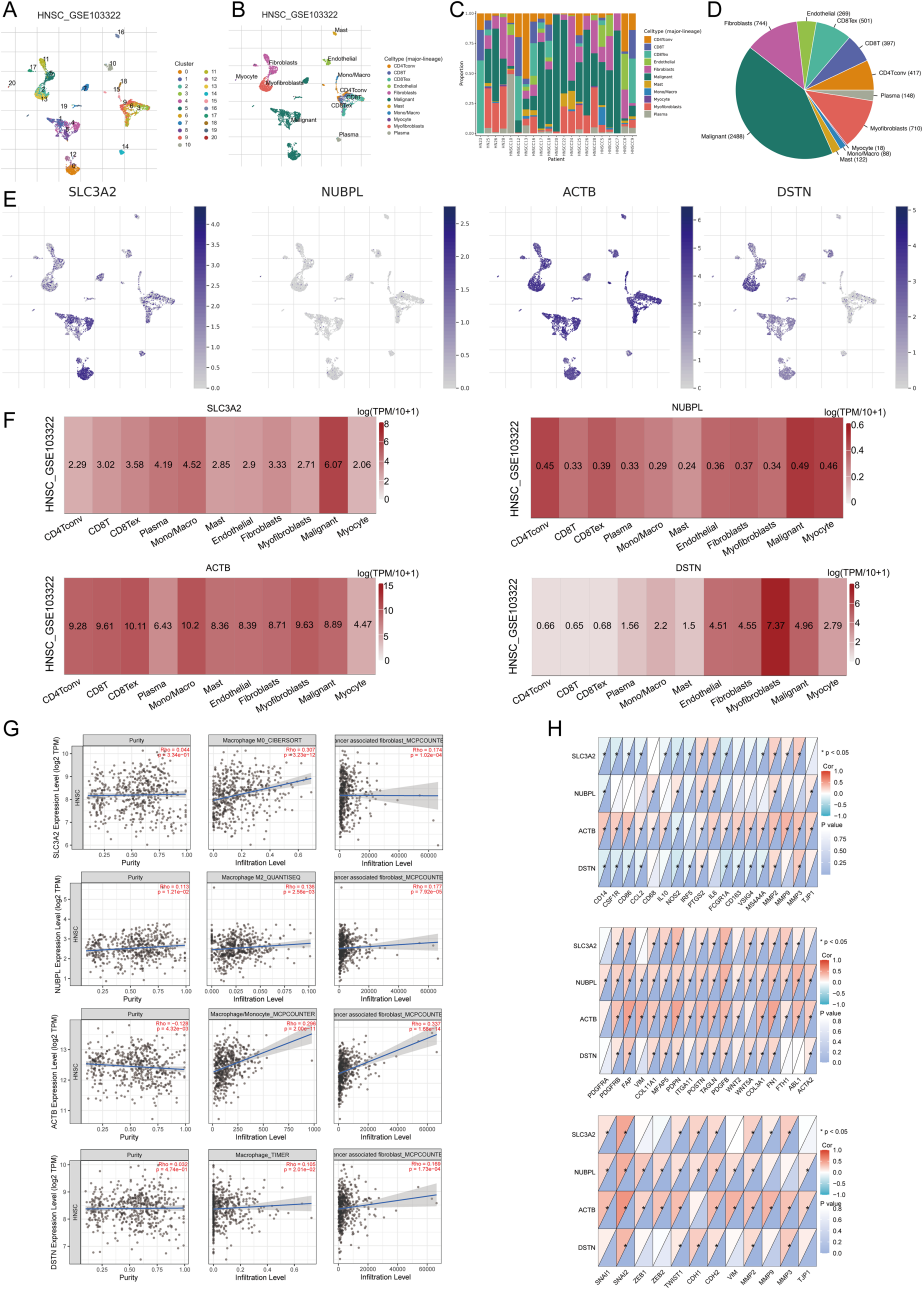
The expression of three prognostic DRGs in different immune cell types in HNSCC. **(A)** Cluster diagram of cell types in scRNA seq data. **(B)** Annotation of different immune cell lineages (HNSCC_GSE103322) in HNSCC tissues. **(C)** The percentage of each cell subtype in different patients. **(D)** The pie chart shows the percentage of each cell. **(E)** Characteristic maps of four prognostic DRGs obtained from scRNA-seq data. **(F)** Heat maps of three prognostic DRGs obtained from scRNA-seq data. **(G)** Correlation between the expression of three prognostic DRGs and macrophages, CAF infiltration as analyzed by TIMER2.0. **(H)** Correlation between the expression of three prognostic DRGs and TAMs, CAF, EMT-related markers. *p<0.05.

In the TISCH database, HNSCC_GSE103322 was divided into 121 cell clusters and 11 cell types, allowing visualization of the distribution and number of various TME-related cells (**Figures 12A, B**). **Figure 12C** shows the percentage of each cell subtype in different patients. The pie chart indicates that macrophages (Mono/Macro) are the most abundant cell type in HNSCC_GSE103322 (**Figure 12D**). We used the HNSCC single cell GSE dataset (HNSCC_GSE103322) to evaluate the expression levels of SLC3A2, NUBPL, ACTB, and DSTN at a single cell level (**Figure 12E**), including Conventional CD4 T cells, CD8 T cells, Exhausted CD8 cells, plasma cells, monocytes or macrophages, mast cells, endothelial cells, fibroblasts, myofibroblasts, malignant cells, and myocytes. It was found that SLC3A2, NUBPL, ACTB, and DSTN were strongly expressed in fibroblasts, Mono/Macro, and malignant cells (**Figure 12F**). Immune infiltration analysis showed a correlation between SLC3A2, NUBPL, ACTB, and DSTN expression and CAF and macrophage infiltration (**Figures 12G**). Combining the above biological function enrichment and immune cell infiltration analysis results, we further explored the association between DRGs and cancer-associated fibroblasts (CAFs), tumor-associated macrophages (TAMs), and related biomarkers. The results showed extensive correlations between CAFs (PDGFRA, PDGFRB, S100A4, FAP, VIM, COL11A1, MFAP5, PDPN, ITGA11, POSTN, TAGLN, PDGFB, WNT2, COL3A1, FGF10, FN1, ABL1, AQP1, ACTA2) and TAMs (CD14, CSF1R, CD86, CCL2, CD68, IL10, NOS2, IRF5, PTGS2, IL6, FCGR1A, CD163, VSIG4, MS4A4A, MMP2, MMP9, MMP3, TJP1) biomarkers. We also analyzed the impact of prognostic DRG expression on EMT and their correlation with EMT-related biomarkers (SNAI1, SNAI2, ZEB1, ZEB2, TWIST1, CDH1, CDH2, VIM, MMP2, MMP9, MMP3), finding significant associations (**Figure 12H**). These results suggest that EMT mediated by prognostic DRGs may be related to fibroblast activation.

### 3.11 Pan-RNA epigenetic modification-related gene expression

In this study, we investigated whether DRG expression is related to pan-RNA epigenetic modification by analyzing the differential expression of pan-RNA epigenetic modification-related genes between high and low risk groups. The results showed significant differences in m6A, m5C, m1A, and m7G modification genes between the two groups (P < 0.01), with high expression in the high-risk group (**Supplementary Figure 10A**). The correlation between these prognostic DRGs and pan-RNA epigenetic modification-related gene expression was analyzed using the TCGA dataset. The results showed significant correlations between the four prognostic DRGs and m6A, m5C, m1A, and m7G modification genes (**Supplementary Figure 10B**). We found that all four prognostic DRGs were positively correlated with highly expressed EIF4E, IGF2BP3, FTO, IFIT5, IGF2BP1, LARP1, NCBP2L, NUDT10, and NUDT11 (**Supplementary Figure 10C**), which were significantly correlated with HNSCC prognosis. These results suggest that DRG expression is closely related to RNA methylation modification in HNSCC.

### 3.12 DNA methylation analysis

Using the GSCA tool, we found that SLC3A2, NUBPL, ACTB, and DSTN expression were significantly negatively correlated with their methylation levels in HNSCC (**Supplementary Figure 11A**). Additionally, in patients classified by age, gender, race, smoking status, nodal metastasis status, tumor grade, individual cancer stage, and TP53 mutation, NUBPL DNA methylation levels were further reduced (**Supplementary Figure 11B**). Thus, decreased DNA methylation levels of DRGs may be potential indicators reflecting the clinical and pathological characteristics of HNSCC patients. We obtained methylation maps of SLC3A2, NUBPL, ACTB, and DSTN from the MethSurv database, presented in a heatmap (**Supplementary Figure 11C**), identifying 60 CpG sites with multiple CpG sites of DRGs showing low methylation in HNSCC patient samples. We further evaluated the prognostic value of each CpG site’s methylation and found 11 CpG sites significantly associated with prognosis (**Supplementary Figures 11D, E**), including cg10922289, cg13402055, cg13109558, cg23551132, cg02356111, cg07476653, cg09041756, cg13677897, cg27056436, cg10573932, and cg19765886. These results indicate that DNA methylation of DRGs is closely related to the development and prognosis of HNSCC.

### 3.13 Prediction and verification of upstream key miRNA

First, we obtained 13 pairs of SLC3A2-miRNA, 8 pairs of NUBPL-miRNA, 13 pairs of ACTB-miRNA, and 42 pairs of DSTN-miRNA by intersecting the ENCORI, miRTarBase, RNA22, RNAInter, and miRWalk databases (**Supplementary Figure 12A**). The potential miRNA gene network was constructed using Cytoscape software (**Supplementary Figure 12B**). We hypothesized that a negative correlation should be observed between the predicted mRNA-miRNA interactions based on the classical role of miRNA in the negative regulation of gene expression. Using the Pan-cancer subproject of the ENCORI database, we screened these candidate miRNA expression correlations in HNSCC. The results showed significant negative correlations between 4 pairs of ACTB-miRNA and 6 pairs of DSTN-miRNA (**Supplementary Figure 13**). Theoretically, miRNAs that strongly bind to ACTB and DSTN should be down-regulated in HNSCC and show poor prognosis. The prognostic effect and expression levels of these potential miRNAs in HNSCC were further verified by Kaplan–Meier plotter and the TCGA database. The results showed that low expression levels of hsa-let-7c-5p, hsa-miR-23b-5p, and hsa-miR-181c-5p were significantly associated with poor prognosis (**Supplementary Figure 12C**), and their expression levels in HNSCC tissues were also significantly lower than in normal tissues (**Supplementary Figure 12D**). Combining the results of negative correlation, survival rate, and expression level analysis, hsa-let-7c-5p, hsa-miR-181c-5p, and hsa-miR-23b-5p were finally confirmed as potential prognostic miRNAs in HNSCC. These results suggest that the ACTB-hsa-let-7c-5p, DSTN-hsa-miR-181c-5p, and DSTN-hsa-miR-23b-5p pathways are key mediators in the occurrence and development of HNSCC and are related to patient prognosis.

### 3.14 Prediction and validation of key miRNAs and potential LncRNAs

**Figure 13:**
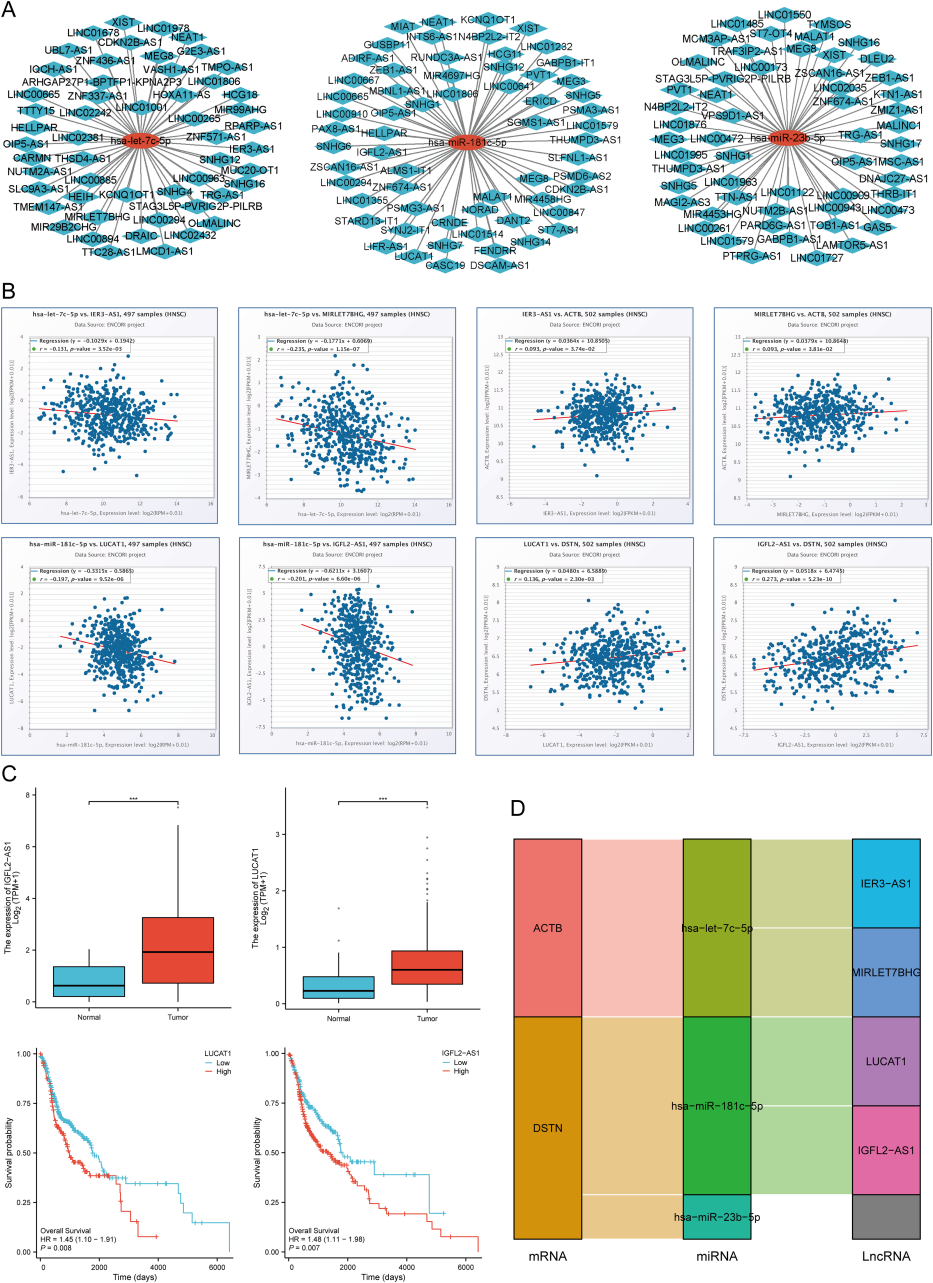
Screening of the LncRNA-miRNA-DRGs regulating axis in HNSCC. **(A)** Prediction of the potential miRNA-lncRNA network through miRNet database. **(B)** Correlation of the potential LncRNAs with hsa-let-7c-5p, hsa-miR-181c-5p, and ACTB, DSTN in HNSCC. **(C)** The expression level and prognostic value of the potential LncRNA (LUCAT1, IGFL2-AS1) in HNSCC. **(D)** The triple regulatory network of mRNA-miRNA-lncRNA. ***p<0.001.

We predicted the upstream lncRNA targets of miRNAs to construct the miRNA-lncRNA axis. The MiRNet database was used to predict lncRNAs, including 53 lncRNAs targeting hsa-let-7c-5p, 62 lncRNAs targeting hsa-miR-181c-5p, and 56 lncRNAs targeting hsa-miR-23b-5p. For better visualization, the miRNA-lncRNA regulation network was established using Cytoscape software (**Figure 13A**). According to the ceRNA hypothesis, lncRNAs can increase mRNA expression by competitively binding to miRNAs. Therefore, lncRNAs were negatively correlated with miRNAs or positively correlated with mRNAs. The correlation between lncRNAs and hsa-let-7c-5p, hsa-miR-181c-5p, and hsa-miR-23b-5p expression was detected using the ENCORI database. It was found that two lncRNAs (IER3-AS1 and MIRLET7BHG) were significantly correlated with hsa-let-7c-5p and ACTB, while two lncRNAs (LUCAT1 and IGFL2-AS1) were significantly correlated with hsa-miR-181c-5p and DSTN (**Figure 13B**). Subsequently, the prognostic value and expression level of these lncRNAs in HNSCC were detected using the TCGA-HNSCC dataset. The results of survival analysis and expression analysis showed that LUCAT1 and IGFL2-AS1 were significantly upregulated in HNSCC, and their upregulation was related to a poor prognosis for HNSCC patients (**Figure 13C**). Finally, we established a key mRNA-miRNA-lncRNA triple regulatory network, which included two mRNAs (ACTB and DSTN), three miRNAs (hsa-let-7c-5p, hsa-miR-181c-5p, and hsa-miR-23b-5p), and four lncRNAs (IER3-AS1, MIRLET7BHG, LUCAT1, and IGFL2-AS1) (**Figure 13D**).

### 3.15 Validation of prognostic analysis with clinical tissue samples

**Figure 14:**
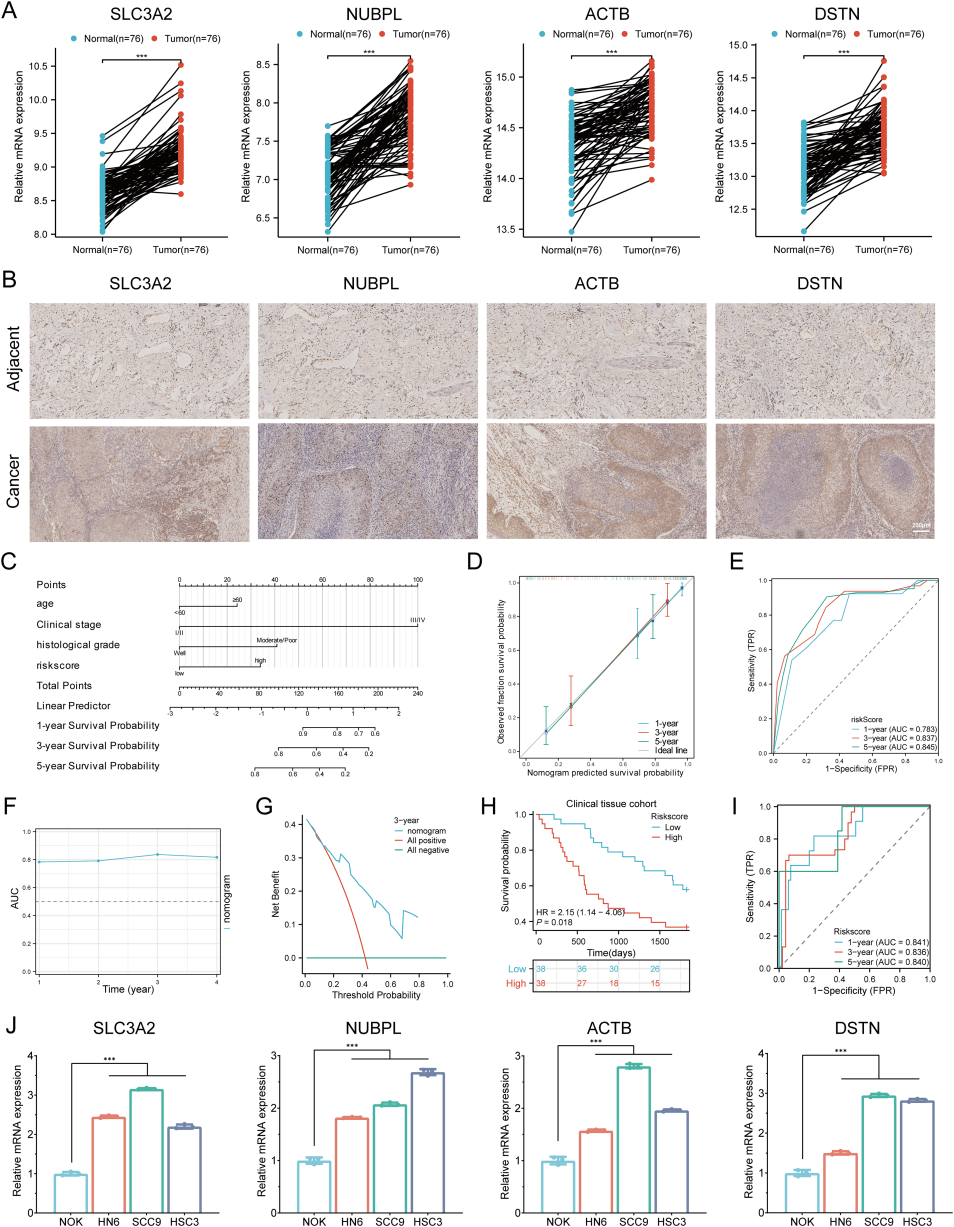
Validation of the prognostic DRGs expression. **(A)** Relative expression of the four prognostic DRGs in adjacent normal tissues and HNSCC tissues. **(B)** Immunohistochemistry analysis of the protein expression levels of four genes in HNSCC and adjacent tissues. **(C)** Nomogram for predicting 1-, 3-, and 5-year OS of clinical HNSCC tissue samples. **(D)** Calibration curve of the nomogram in the external validation group. **(E)** ROC curves for predicting 1-, 3-, and 5-year OS in the external validation group. **(F)** Time-dependent AUC curve shows the nomogram to predict OS performance in the external validation group. **(G)** DCA curves for the nomogram in the external validation group. **(H)** Overall survival curve of HNSCC patients in high/low-risk groups. **(I)** Time-dependent ROC curve for 1-, 3-, and 5-year OS for DRGs. **(J)** Differential expression of four prognostic DRGs in NOK, HN6, SCC9, and HSC3 cell lines. ***p < 0.001.

To verify the mRNA-level analysis results from the TCGA, we used RT-qPCR to validate the expression patterns of the four prognostic DRGs in 76 HNSCC clinical specimens, comparing HNSCC tissues with adjacent non-tumor tissues. We found that SLC3A2, NUBPL, ACTB, and DSTN were significantly upregulated in HNSCC tissues compared to adjacent tissues (**Figure 14A**). Based on qPCR validation results, we performed immunohistochemical staining on HNSCC tissues and adjacent normal tissues to verify the protein expression of the four prognostic DRGs in clinical specimens. The results showed that SLC3A2, NUBPL, ACTB, and DSTN proteins were significantly elevated in HNSCC tissues compared to adjacent tissues (**Figure 14B**). To fully understand the clinical significance of the DRG risk score, we analyzed multiple subgroups, including Age, Gender, distant metastasis, Smoking, Alcohol, N stage, Clinical stage, tumor site, histological grade, and Treatment. The results showed that the risk score was significantly associated with Clinical stage and histological grade in HNSCC patients (P < 0.05, **Supplementary Table 6**). To assess the independent predictive value of the prognostic model for our HNSCC clinical samples, we conducted univariate and multivariate Cox regression analyses on risk scores and other clinical features. We found that N stage (P = 0.031, HR = 0.113 (0.015 - 0.820)), Clinical stage (P = 0.001, HR = 0.140 (0.043 - 0.454)), histological grade (P = 0.026, HR = 2.146 (1.097 - 4.198)), and Risk score (P = 0.004, HR = 2.523 (1.349 - 4.719)) showed good prognostic value in univariate Cox regression analysis (P < 0.05). In multivariate analysis, Age (P = 0.010, HR = 2.530 (1.243 - 5.148)), Clinical stage (P = 0.004, HR = 0.151 (0.041 - 0.551)), histological grade (P = 0.022, HR = 2.269 (1.124 - 4.581)), and Risk score (P = 0.023, HR = 2.232 (1.115 - 4.471)) were identified as independent prognostic indicators for HNSCC patients (**Supplementary Table 7**). Based on the above multivariate Cox analysis, we combined these independent prognostic factors to construct a nomogram for predicting short-term and long-term survival rates in HNSCC patients. The nomogram was externally validated using the clinical HNSCC tissue sample cohort. The C-index of the nomogram was 0.766 (0.730-0.802) (**Figure 14C**). Calibration curves showed satisfactory consistency between predicted and observed results (**Figure 14D**). The 1-, 3-, and 5-year AUCs of the ROC curves were 0.783, 0.837, and 0.845, respectively (**Figure 14E**). Time-dependent AUC curves demonstrated the nomogram’s performance in predicting OS in the clinical sample validation cohort (**Figure 14F**). DCA confirmed the clinical utility of the nomogram in predicting survival rates (**Figure 14G**). All these results were consistent, indicating that the DRG prognostic model performs well in predicting the prognosis of HNSCC patients. Based on the predictive efficiency of the prognostic model constructed from the TCGA-HNSCC dataset, we validated the model’s efficiency using HNSCC clinical tissue samples from our hospital. Using the same formula to calculate the risk scores, HNSCC patients in the clinical cohort were divided into high-risk and low-risk groups based on the median value. Survival analysis showed that patients with higher risk scores had shorter OS than those with lower risk scores (**Figure 14H**, p = 0.018, HR = 2.15 (1.14 - 4.06)), consistent with the results from the TCGA and GEO cohorts. The 1-, 3-, and 5-year AUCs of the ROC curves were 0.841, 0.836, and 0.840, respectively (**Figure 14I**). Additionally, we explored the mRNA expression of SLC3A2, NUBPL, ACTB, and DSTN in HNSCC cell lines. Consistent with tissue expression levels, the mRNA expression of these genes was significantly upregulated in HNSCC cell lines (NH6, HSC3, and SCC9) compared to normal human epithelial cells (NOK) (**Figure 14J**). Therefore, all these results consistently confirmed the predictive efficiency of the constructed prognostic model, indicating its reliability and validity in predicting the prognosis of HNSCC patients.

### 3.16 *In vitro* cell experiment of DSTN in HNSCC

**Figure 15:**
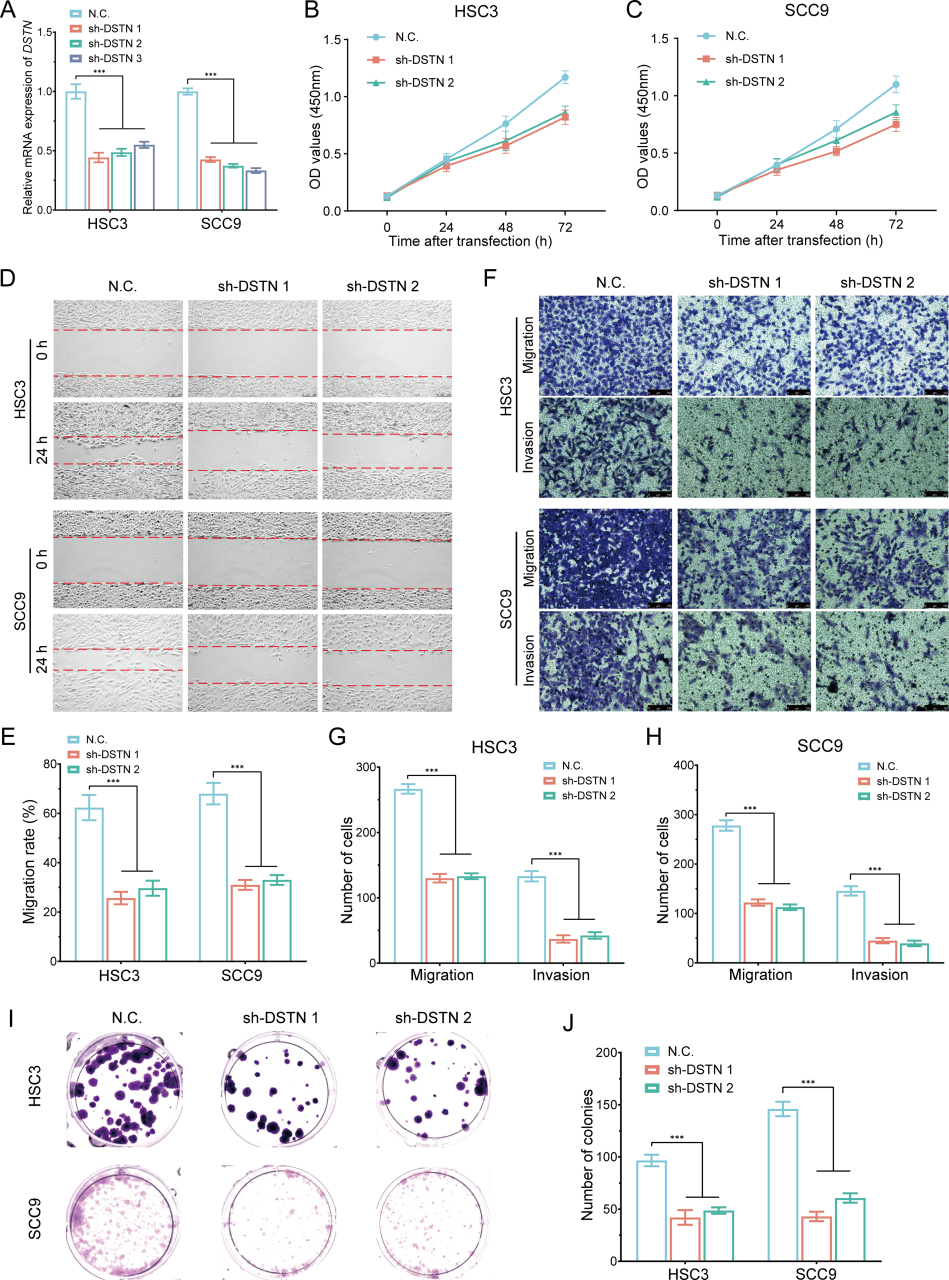
*In vitro* cell experiment of DSTN in HNSCC. **(A)** RT-qPCR analysis showing the knockout efficiency of DSTN in HSC3 and SCC9 cells. **(B, C)** CCK-8 assays were performed in stable HSC3 and SCC9 cells with DSTN knockdown. **(D, E)** Wound-healing assays in stable HSC3 and SCC9 cells with DSTN knockdown. **(F-H)** Transwell migration and invasion assays in stable HSC3 and SCC9 cells with DSTN knockdown. **(I, J)** Clone formation experiment following DSTN knockdown in HSC3 and SCC9 cells. ***p < 0.001.

To further investigate the role and functional significance of DRGs in HNSCC, we conducted DSTN gene knockout experiments in HSC3 and SCC9 cells (**Figure 15A**). Following DSTN gene knockout, CCK-8 assays showed that the proliferation rate of HSC3 and SCC9 cells was significantly reduced (**Figures 15B, C**). Wound healing assays and migration invasion experiments indicated that the migration (**Figures 15D, E**) and invasion abilities (**Figures 15F–H**) of HSC3 and SCC9 cells were significantly decreased. Colony formation assays showed that DSTN gene knockout significantly inhibited the proliferation of HSC3 and SCC9 cells (**Figures 15I, J**). In summary, the inhibition of cell proliferation following DSTN gene knockout suggests that DSTN plays a critical role in the development of HNSCC.

## 4 Discussion

Head and neck squamous cell carcinoma (HNSCC) is a highly aggressive cancer characterized by significant heterogeneity and immunosuppression. The prognosis and treatment strategies are closely related to the diagnosis and therapeutic options. In recent years, disulfidptosis, a specific form of cell death, has attracted attention for its potential role in cancer development. Understanding the role of disulfidptosis in HNSCC is crucial for elucidating the biological mechanisms of the tumor, improving patient prognosis, and developing new therapeutic strategies. This type of research may uncover new biomarkers that support personalized treatment, especially in the field of immunotherapy. The study employed a variety of methods, including gene expression analysis, proteomics research, and statistical analysis of clinical data. Researchers collected numerous HNSCC patient samples from multiple databases, utilized advanced bioinformatics tools for in-depth analysis, and performed external validation using clinical tissue samples.

In our study, we investigated 24 existing DRGs and identified two disulfidptosis-related subtypes. Through DEGs between the two subtypes, we found that DRGs are involved in multiple signaling pathways. Literature has reported that these pathways are closely related to tumor invasion and metastasis. These pathways include ECM-receptor interaction (41), Focal adhesion (42), cGMP-PKG signaling pathway (43), TGF-beta signaling pathway (44), PI3K-Akt signaling pathway (45), MAPK signaling pathway (46), ERBB signaling pathway (47), EGFR Signaling Pathway (48), B Cell Receptor Signaling Pathway (49), VEGFR1 Pathway, and Wnt Signaling Pathway (50).

Based on the expression patterns and prognostic analysis of disulfidptosis-related genes, a predictive model was constructed using LASSO Cox regression, identifying four prognostic disulfidptosis-related genes (SLC3A2, NUBPL, ACTB, DSTN). The K-M curve indicated that patients with high-risk scores in this model had poorer prognosis compared to the low-risk group. ROC curves for 1-year, 3-year, and 5-year survival probabilities revealed good specificity and sensitivity of the prognostic model. The TCGA internal and GEO external validation cohorts confirmed the model’s effectiveness and stability in predicting the prognosis of HNSCC patients. Univariate and multivariate Cox analyses identified the model as an independent prognostic factor for HNSCC. We further constructed a prediction nomogram based on the signature to predict clinical outcomes for HNSCC patients. High expression of disulfidptosis-related markers was closely related to poor clinical prognosis.

The construction of prognostic signatures plays a critical role in providing more refined and accurate assessments of prognosis. Several recent studies have developed prognostic models based on the expression of disulfidptosis-related genes (DRGs) in HNSCC. For example, a risk model designed to predict prognosis and immune features in sarcoma patients identified DRGs as independent prognostic factors (9). In a similar vein, a prognostic gene signature based on cuproptosis-related genes demonstrated high predictive accuracy for prognosis in hepatocellular carcinoma, highlighting the applicability of such models across various cancer types (10). These studies emphasize the growing recognition of disulfidptosis and its related genes as valuable prognostic biomarkers in malignancies. In the present study, we validated the expression levels and prognostic significance of DRGs using tissue samples from HNSCC patients treated at our hospital. Compared to adjacent tissues, SLC3A2, NUBPL, ACTB, and DSTN were significantly upregulated in HNSCC tissues, demonstrating high sensitivity and specificity for HNSCC diagnosis. The constructed DRG prognostic signature showed strong performance in predicting patient outcomes. Analysis of clinical data and DRG expression in HNSCC patients suggests that disulfidptosis may serve as a reliable biomarker for assessing prognosis, offering more accurate predictions of disease progression and treatment response. This is consistent with previous findings where similar DRGs were shown to have prognostic value in hepatocellular carcinoma, further supporting their utility as prognostic markers across different cancer types (11). Additionally, the role of these genes in immune regulation has been confirmed, suggesting their potential to predict the efficacy of immunotherapy in cancers such as HNSCC (9).

Four prognostic disulfidptosis-related genes (SLC3A2, NUBPL, ACTB, DSTN) were identified as prognostic markers in this study. Research indicates that these marker genes are closely related to tumors. Specifically, Solute Carrier Family 3 Member A2 (SLC3A2) is an important member of the solute carrier family, involved in the regulation of amino acid transport proteins and mediating this exchange process. Studies have shown that high expression of SLC3A2 is closely related to the growth, invasion, and metastasis of various malignancies, such as lung adenocarcinoma and colorectal cancer (51). Nucleotide Binding Protein - Like (NUBPL) is an assembly factor of human mitochondrial complex I and the largest member of the mitochondrial respiratory chain. Data suggest that NUBPL promotes the migration and invasion of colorectal cancer cells by inducing EMT and activating ERK (52). β-Actin (ACTB) is a highly conserved cytoskeletal structural protein considered a common housekeeping gene and widely used as a control for measuring the expression of various diseases. However, ACTB is abnormally expressed in various cancers, altering the cytoskeleton and affecting tumor invasiveness and metastasis (53). DSTN, a key actin-binding protein, plays a significant role in actin dynamics and cell migration. DSTN’s expression is modulated by factors in the tumor microenvironment, impacting tumor progression and invasion (54, 55).

In head and neck squamous cell carcinoma (HNSCC), genetic variations can modulate the immune system’s response to cancer cells, while disulfidptosis-related processes may influence the activity and distribution of immune cells within the tumor microenvironment. Recent studies have emphasized the impact of immune-related processes on prognosis, supporting our findings of a significant correlation between disulfidptosis-related genes (DRGs) and immune cell infiltration (10). The growing body of research on the copy number variation (CNV) of DRGs and its effect on the immune microenvironment has provided valuable insights into how epigenetic modifications can shape tumor immunity, underscoring the potential of DRGs as therapeutic targets for immune modulation (11). Although current research has advanced our understanding of the role of disulfidptosis in HNSCC, many critical questions remain unresolved. Future studies, as suggested by recent reports (9), should focus on validating the predictive accuracy of disulfidptosis-related prognostic models in larger clinical cohorts. Additionally, it will be important to explore how genetic variations and DNA methylation contribute to tumor progression and treatment response. Addressing these challenges will be crucial for translating current findings into clinical practice, particularly for diverse patient populations and different cancer subtypes.

We utilized multiple bioinformatics tools and algorithms to analyze and interpret complex genomic data. Additionally, we explored the correlation between DRG expression and patient immune phenotypes, validating DRG expression using clinical samples through PCR, immunohistochemistry, and cell line experiments. These efforts provide new insights into tumor immune escape mechanisms and contribute to the development of novel immunoregulatory strategies. Our research revealed a significant correlation between prognostic DRGs and the abundance of certain immune cells, such as B cells, T regulatory cells (Tregs), M2 macrophages, and neutrophils. This immune cell infiltration was associated with improved clinical prognosis in HNSCC patients. Moreover, using the TIMER database, we found that the expression of NUBPL and ACTB was closely related to tumor purity, while the correlation of SLC3A2 and DSTN with tumor purity was less pronounced. ACTB (β-actin) is a cytoskeletal protein commonly used as a marker for cell expression, and its expression may more strongly reflect the quantity and activity of tumor cells. NUBPL, on the other hand, may be associated with the metabolic state, proliferation, or survival of tumor cells. In contrast, DSTN, an actin-related protein involved in cytoskeletal remodeling and cell migration, may be influenced by multiple factors in the tumor microenvironment, not just by the proportion of tumor cells. Therefore, DSTN expression might not entirely depend on changes in tumor purity, especially in tumor samples with multiple cell populations. The presence of immune cells, fibroblasts, and other non-tumor components may modulate DSTN expression. Consequently, DSTN could play a crucial role in tumor cell migration, invasion, and interactions with other cell types, with its expression pattern potentially having a weaker relationship with tumor purity due to dynamic regulation by the tumor microenvironment. Most current research focuses primarily on T cell immunity, with increasing evidence supporting the beneficial role of B cell infiltration in the survival of HNSCC patients (56, 57). In contrast, increased neutrophil infiltration and a higher neutrophil-to-lymphocyte ratio are associated with poor prognosis in HNSCC patients (58, 59). M2 macrophages are known to promote tumor growth, invasion, and metastasis within the tumor microenvironment (60). Recent studies have confirmed that high levels of macrophage infiltration in the TME are significantly correlated with poor prognosis in HNSCC patients (61). Therefore, research into treatment strategies targeting these immune cells holds significant clinical value.

In the tumor microenvironment (TME) of HNSCC, the relationship between disulfide cell death and other modes of cell death (such as apoptosis, necrosis, pyroptosis, ferroptosis, and autophagy) is complex (62). Since different modes of cell death share many key molecules, the epigenetic modifications of these molecules can influence various types of cell death (63). Reactive oxygen species (ROS) play a pivotal role in regulating different forms of cell death. Excessive ROS can induce disulfide cell death and may also promote ferroptosis (64), autophagy (65), pyroptosis (66), copper death (67), and apoptosis (68) through mitochondrial damage and DNA damage. Additionally, tumor-associated immune cells (such as tumor-associated macrophages [TAMs] and myeloid-derived suppressor cells [MDSCs]) influence the activation of different cell death pathways by secreting inflammatory factors and modulating the redox environment (69). Metabolic abnormalities in HNSCC cells, such as lactate accumulation, may alter the intracellular redox balance (70), determining the preferential activation of disulfide cell death or other forms of cell death. Therefore, regulating oxidative stress and metabolic state in the HNSCC microenvironment can affect tumor cell sensitivity to treatments (such as radiotherapy, chemotherapy, and immunotherapy). DRGs may serve as novel therapeutic targets, and their combination with other cell death pathways could enhance treatment efficacy.

Recent studies have shown that immunosuppressive cells in the tumor microenvironment may hinder anti-tumor immunotherapy, leading to failure in cancer immunotherapy (71). TAMs are important immune cells in the tumor microenvironment, playing a significant role in the progression of many tumors, with M2-like macrophages being the predominant phenotype (72). Chaudhari et al. (73) found that the CD163 TAM score in oral squamous cell carcinoma was significantly positively correlated with higher tumor stage, lymph node metastasis, and tumor progression. Additionally, TAMs can induce EMT in tumor cells, promoting HNSCC invasion and metastasis and being associated with poor prognosis (74). CAFs are the most abundant stromal cells in tumors, playing a crucial role in tumorigenesis, development, metastasis, and drug resistance. CAFs can secrete various signaling molecules, such as TGF-β, Wnt, and Notch, which can activate the EMT process and promote tumor cell invasion and metastasis (75). This study used scRNA-seq and bioinformatics techniques to reveal the close correlation between prognostic DRGs and TAMs, and CAFs, EMT-related markers. However, in this study, we found that high infiltration levels of Macrophages.M2 cells in HNSCC were associated with good prognosis. Therefore, the mechanisms by which DRGs and immune cell phenotypes affect the prognosis of HNSCC patients may require more evidence and discussion.

Currently, immunotherapy, particularly the use of immune checkpoint inhibitors, has brought new hope to HNSCC treatment, but not all patients benefit from it. In this study, we explored the correlation between DRG expression and immune checkpoint genes and found that CD274, PDCD1LG2, and SIGLEC15 were highly expressed in the C1 group, and PDCD1LG2, SIGLEC15 were higher in the high-risk group. We also found that the high-risk group had higher TIDE scores, suggesting that patients with lower risk scores might benefit from ICI therapy. We collected four external independent immune therapy cohorts (anti-PD-1/PD-L1/CTLA-4) and a clinical cohort of advanced HNSCC patients receiving immune therapy to evaluate the performance of DRGs in predicting immune therapy response. The results indicated that DRGs have good predictive ability for immune response in patients, with the low-risk group being more suitable for immune therapy. We also found that high TMB, MSI, and mRNAsi groups had poor prognosis for HNSCC patients and were more prone to progression. Additionally, prognostic DRGs were positively or negatively correlated with various chemotherapeutic and targeted drugs, but further experiments are needed to verify this. Therefore, these results provide new potential therapeutic targets for HNSCC treatment.

In our study, DSTN was found to be significantly associated with tumor progression and showed higher expression levels in head and neck squamous cell carcinoma (HNSCC). As a protein involved in cytoskeletal remodeling, DSTN has been confirmed in several studies to play a critical role in tumor cell migration, proliferation, and invasion, particularly during tumor metastasis. Its key role in our computational model makes DSTN a candidate gene for validation. Further survival analysis revealed that DSTN was significantly correlated with overall survival (OS), progression-free survival (PFS), and disease-specific survival (DSS), with P-values of 0.02, 0.015, and 0.02, respectively, further supporting its close association with tumor prognosis. Immune infiltration analysis and transcriptomic data also indicated that DSTN is closely related to immune cell infiltration in the tumor microenvironment and tumor progression, reinforcing its reliability as a potential oncogene. Cellular experiments showed that DSTN was highly expressed in multiple cell lines, and clinical tissue samples revealed significantly higher DSTN expression in tumor tissues compared to normal tissues and other candidate genes. We also found that DSTN exhibited stronger associations with pathways such as hsa-miR-181c-5p/LUCAT1, IGFL2-AS1, and hsa-miR-23b-5p, suggesting its significant role in tumor regulation. Based on this evidence, we selected DSTN as the key gene for validation.

In further experiments, we validated the effect of DSTN gene knockout on two HNSCC cell lines. The results showed that DSTN knockout significantly reduced cell proliferation, migration, and invasion, providing further evidence for its potential as a prognostic marker in HNSCC. High DSTN expression is closely associated with tumor cell proliferation. Studies have shown that DSTN regulates cell cycle-related proteins to promote cell cycle progression and drive tumor cell proliferation (76). Additionally, DSTN enhances tumor cell proliferation by modulating the β-catenin pathway. Zhang HJ et al. (77) found that DSTN promotes the nuclear translocation of β-catenin and induces epithelial-mesenchymal transition (EMT), increasing the malignancy of lung cancer. DSTN is closely linked to EMT, a key process through which tumor cells acquire the ability to migrate and invade. DSTN interacts with the cytoskeleton to promote the EMT process in tumor cells, enhancing their migratory capacity, and drives this process by regulating markers such as N-cadherin and Vimentin (78, 79). Furthermore, DSTN influences tumor microenvironment remodeling by interacting with cancer-associated fibroblasts (CAFs), further promoting tumor invasion and metastasis (80–82). DSTN not only plays a role in actin remodeling but may also regulate tumor cell migration and invasion by activating key signaling pathways such as the Rho family GTPases (83). Research by Wen R et al. (84) suggests that DSTN knockdown enhances colorectal cancer cell sensitivity to radiation therapy, while DSTN overexpression confers resistance to radiation and enhances the malignant characteristics of tumor cells through activation of the Wnt/β-catenin signaling pathway.

Additionally, we explored the regulatory axis of DSTN/hsa-miR-181c-5p/LUCAT1 and IGFL2-AS1 in HNSCC, which may be involved in tumor invasion and metastasis. Increasing evidence suggests that the dysregulation of long non-coding RNAs (lncRNAs) plays a crucial role in the pathogenesis of various cancers, particularly in cell proliferation and apoptosis. Lung cancer-associated transcript 1 (LUCAT1) was first identified as being related to smoking-associated lung cancer, and studies have shown that LUCAT1 promotes the development of laryngeal cancer by targeting and inhibiting miR-493 (85). Abnormal expression of LUCAT1 affects glioma cell biology by regulating ABCB1 and promoting the activation of the RAS pathway (86). Moreover, IGFL2-AS1 is highly expressed in several cancers, promoting tumor progression by influencing cell proliferation, migration, and EMT (87). Abnormal expression of IGFL2-AS1 enhances the proliferation, migration, and invasion of colorectal cancer cells and is associated with poor patient prognosis (88). Additionally, it is closely related to radioresistance in colorectal cancer (89). While current research provides significant insights into the role of disulfidptosis in HNSCC, several questions remain. First, the disulfidptosis-related prognostic model was constructed using the TCGA database and validated through internal TCGA cohorts, external GEO cohorts, and clinical sample data from our hospital, demonstrating consistent predictive performance for HNSCC prognosis. However, larger clinical cohorts are needed to validate the predictive accuracy of the disulfidptosis-related prognostic model. Second, the precise mechanisms by which disulfidptosis influences HNSCC development require further investigation. Lastly, translating these findings into clinical practice, especially in different populations and cancer subtypes, necessitates additional research and clinical trials.

Despite the potential role of disulfidptosis-related genes in HNSCC revealed through bioinformatics analysis and experimental validation, there are still limitations, including insufficient sample size, unclear functional mechanisms, and a lack of clinical application validation. Future studies should increase clinical sample sizes to further confirm the accuracy of DRGs as prognostic markers and explore the mechanisms underlying DRGs in HNSCC, particularly their impact on the tumor microenvironment. Moreover, translating these findings into clinical practice, especially regarding their effectiveness in different populations and cancer subtypes, requires more research and clinical trials.

## 5 Conclusion

This study is the first to elucidate the important role of disulfidptosis in the development, clinical prognosis, and immunotherapy response of HNSCC. Based on four disulfidptosis-related genes, a prognostic model for predicting the survival of HNSCC patients and a potential mRNA-miRNA-lncRNA regulatory network were constructed, providing a new perspective for HNSCC prognosis research. Additionally, the disulfidptosis gene DSTN has been experimentally proven to be a key gene in promoting HNSCC progression by enhancing tumor cell proliferation, migration, and invasion. Its potential DSTN/hsa-miR-181c-5p/LUCAT1, IGFL2-AS1 regulatory network may serve as a novel therapeutic target.

## Data Availability

The datasets are available in TCGA database (https://portal.gdc.cancer.gov/), GDSC database (https://www.cancerrxgene.org/), GeneMANIA (http://www.genemania.org), GSEA (http://software.broadinstitute.org/gsea/index.jsp), Human Protein Atlas database (https://www.proteinatlas.org), cBioPortal (http://www.cbioportal.org/), GDSC database (https://www.cancerrxgene.org/), TISIDB (http://cis.hku.hk/TISIDB), TISCH database (http://tisch.comp-genomics.org/), ENCORI database (http://starbase.sysu.edu.cn/), RNAInter database (http://www.rnainter.org/), miRNet database (http://www.mirnet.ca/), RNA22 database (https://cm.jefferson.edu/rna22/interactive) as well as TIMER database (https://cistrome.shinyapps.io/timer/).
