# Supplementary material for: Disulfidptosis-related gene signatures as prognostic biomarkers and predictors of immunotherapy response in HNSCC
Source: Front Immunol. 2025 Jan 17;15:1456649. doi: 10.3389/fimmu.2024.1456649 (PMC11782277; doi:10.3389/fimmu.2024.1456649)
Supplement: Supplementary file 1 [file DataSheet1.zip › Supplementary Table 7.docx]

**Supplementary Table 7. Univariate and multivariate Cox regression analysis for clinical variables in HNSCC.**

| Characteristics | Univariate analysis | |  | Multivariate analysis | |
| --- | --- | --- | --- | --- | --- |
|  | Hazard ratio (95% CI) | P value |  | Hazard ratio (95% CI) | P value |
| Age (<60 vs. ≥60) | 1.953 (0.999 - 3.819) | 0.050 |  | 2.530 (1.243 - 5.148) | **0.010** |
| Gender (Female vs. Male ) | 1.170 (0.575 - 2.379) | 0.666 |  |  |  |
| distant metastasis (M0 vs. M1) | 2.310 (0.710 - 7.513) | 0.164 |  |  |  |
| Smoking (No vs. Yes ) | 1.027 (0.546 - 1.932) | 0.934 |  |  |  |
| Alcohol (Yes vs. No) | 0.686 (0.289 - 1.630) | 0.393 |  |  |  |
| N stage (N0 vs. N1/2/3) | 0.113 (0.015 - 0.820) | **0.031** |  | 0.470 (0.049 - 4.475) | 0.511 |
| Clinical stage (I/II vs. III/IV) | 0.140 (0.043 - 0.454) | **0.001** |  | 0.151 (0.041 - 0.551) | **0.004** |
| tumor site (Oropharynx/nasopharynx vs. Cavum Oris/Larynx) | 1.688 (0.916 - 3.111) | 0.093 |  | 0.730 (0.365 - 1.462) | 0.375 |
| histological grade (Well vs. Moderate/Poor) | 2.146 (1.097 - 4.198) | **0.026** |  | 2.269 (1.124 - 4.581) | **0.022** |
| Treatment (mono vs. multi) | 0.370 (0.114 - 1.198) | 0.097 |  | 0.616 (0.179 - 2.115) | 0.441 |
| Riskscore (low vs. high) | 2.523 (1.349 - 4.719) | **0.004** |  | 2.232 (1.115 - 4.471) | **0.023** |
