# Supplementary material for: Disulfidptosis-related gene signatures as prognostic biomarkers and predictors of immunotherapy response in HNSCC
Source: Front Immunol. 2025 Jan 17;15:1456649. doi: 10.3389/fimmu.2024.1456649 (PMC11782277; doi:10.3389/fimmu.2024.1456649)
Supplement: Supplementary file 1 [file DataSheet1.zip › Supplementary Table 1.docx]

**Supplementary Table 1. The abbreviations of DRGs in this study.**

| DRGs Abbreviation | Full name |
| --- | --- |
| SLC7A11 | solute carrier family 7 member 11 |
| SLC3A2 | solute carrier family 3 member 2 |
| RPN1 | ribophorin I |
| NCKAP1 | NCK-associated protein 1 |
| NUBPL | nucleotide binding protein-like |
| NDUFA11 | NADH dehydrogenase (ubiquinone) 1 alpha subcomplex, 11, 14.7kDa |
| LRPPRC | leucine-rich pentatricopeptide repeat containing |
| OXSM | 3-oxoacyl-ACP synthase, mitochondrial |
| NDUFS1 | NADH dehydrogenase (ubiquinone) Fe-S protein 1, 75kDa (NADH-coenzyme Q reductase) |
| GYS1 | glycogen synthase 1 (muscle) |
| ACTN4 | actinin, alpha 4 |
| ACTB | actin, beta |
| CD2AP | CD2-associated protein |
| CAPZB | capping protein (actin filament) muscle Z-line, beta |
| DSTN | destrin (actin depolymerizing factor) |
| FLNA | filamin A, alpha |
| FLNB | filamin B, beta |
| INF2 | inverted formin, FH2 and WH2 domain containing |
| IQGAP1 | IQ motif containing GTPase activating protein 1 |
| MYH10 | myosin, heavy chain 10, non-muscle |
| MYL6 | myosin, light chain 6, alkali, smooth muscle and non-muscle |
| MYH9 | myosin, heavy chain 9, non-muscle |
| PDLIM1 | PDZ and LIM domain 1 |
| TLN1 | talin 1 |
