# Supplementary material for: Disulfidptosis-related gene signatures as prognostic biomarkers and predictors of immunotherapy response in HNSCC
Source: Front Immunol. 2025 Jan 17;15:1456649. doi: 10.3389/fimmu.2024.1456649 (PMC11782277; doi:10.3389/fimmu.2024.1456649)
Supplement: Supplementary file 1 [file DataSheet1.zip › Supplementary Table 2.docx]

**Supplementary Table 2.** Primer sequences of genes

| Real-time quantitative PCR primer sequence | |
| --- | --- |
| **Gene** | Sequence (5’- 3’ on minus strand) |
| *GAPDH* | Fwd: GGAGCGAGATCCCTCCAAAAT |
|  | Rev: GGCTGTTGTCATACTTCTCATGG |
| *SLC3A2* | Fwd: TGAATGAGTTAGAGCCCGAGA |
|  | Rev: GTCTTCCGCCACCTTGATCTT |
| *NUBPL* | Fwd: CTGAGATGTTTCGCAGAGTCC |
|  | Rev: CAAGGGTCTGTGCTAGTTTCC |
| *ACTB* | Fwd: CATGTACGTTGCTATCCAGGC |
|  | Rev: CTCCTTAATGTCACGCACGAT |
| *DSTN* | Fwd: ATTTTGTGGGAATGCTTCCTGA |
|  | Rev: GCATCCTTGGAGCTTGCATAG |
