# Supplementary material for: Disulfidptosis-related gene signatures as prognostic biomarkers and predictors of immunotherapy response in HNSCC
Source: Front Immunol. 2025 Jan 17;15:1456649. doi: 10.3389/fimmu.2024.1456649 (PMC11782277; doi:10.3389/fimmu.2024.1456649)
Supplement: Supplementary file 1 [file DataSheet1.zip › Supplementary Table 3.docx]

**Supplementary Table 3. Gene sets enriched in phenotype high.**

| ID | NES | p.adjust | FDR |
| --- | --- | --- | --- |
| KEGG_WNT_SIGNALING_PATHWAY | 1.402602 | 0.0018 | 0.0012 |
| WP_ERBB_SIGNALING_PATHWAY | 1.515824 | 0.0006 | 0.0004 |
| PID_VEGFR1_PATHWAY | 1.751882 | 0.0014 | 0.0010 |
| KEGG_TGF_BETA_SIGNALING_PATHWAY | 1.479827 | 0.0023 | 0.0016 |
| WP_B_CELL_RECEPTOR_SIGNALING_PATHWAY | 1.424507 | 0.0042 | 0.0028 |
| WP_EGFEGFR_SIGNALING_PATHWAY | 1.630378 | 0.0004 | 0.0003 |
| KEGG_FOCAL_ADHESION | 1.626672 | 0.0004 | 0.0003 |
| WP_PI3KAKT_SIGNALING_PATHWAY | 1.421576 | 0.0004 | 0.0003 |
| WP_HEAD_AND_NECK_SQUAMOUS_CELL_CARCINOMA | 1.553050 | 0.0007 | 0.0005 |
