# Supplementary material for: Disulfidptosis-related gene signatures as prognostic biomarkers and predictors of immunotherapy response in HNSCC
Source: Front Immunol. 2025 Jan 17;15:1456649. doi: 10.3389/fimmu.2024.1456649 (PMC11782277; doi:10.3389/fimmu.2024.1456649)
Supplement: Supplementary file 1 [file DataSheet1.zip › Supplementary Table 4.docx]

**Supplementary Table 4. The clinical characters of HNSCC patients in TCGA cohort**

| Clinical characters | number |
| --- | --- |
| Alive | 286 (56.7%) |
| Dead | 218 (43.3%) |
| Age, median (IQR) | 61 (53, 69) |
| Sex, n (%) |  |
| FEMALE | 134 (26.6%) |
| MALE | 370 (73.4%) |
| Race, n (%) |  |
| WHITE | 430 (87.9%) |
| AMERICAN INDIAN | 2 (0.4%) |
| BLACK | 47 (9.6%) |
| ASIAN | 10 (2%) |
| T, n (%) |  |
| T4 | 180 (36.5%) |
| T3 | 133 (27%) |
| T2 | 146 (29.6%) |
| T1 | 34 (6.9%) |
| N, n (%) |  |
| N0 | 242 (49.9%) |
| N1 | 81 (16.7%) |
| N2 | 155 (32%) |
| N3 | 7 (1.4%) |
| M, n (%) |  |
| M0 | 479 (99%) |
| M1 | 5 (1%) |
| Stage, n (%) |  |
| IV | 307 (60.9%) |
| III | 91 (18.1%) |
| II | 81 (16.1%) |
| I | 25 (5%) |
| Grade, n (%) |  |
| G2 | 301 (62.2%) |
| G3 | 119 (24.6%) |
| G1 | 62 (12.8%) |
| G4 | 2 (0.4%) |
| Smoking, n (%) |  |
| No | 113 (22.9%) |
| Yes | 381 (77.1%) |
| Radiation, n (%) |  |
| NO | 63 (34.2%) |
| YES | 121 (65.8%) |
| Neoadjuvant, n (%) |  |
| No | 494 (98%) |
| Yes | 10 (2%) |
| Therapy, n (%) |  |
| Chemotherapy | 159 (98.8%) |
| Immunotherapy | 2 (1.2%) |
