# Supplementary material for: Disulfidptosis-related gene signatures as prognostic biomarkers and predictors of immunotherapy response in HNSCC
Source: Front Immunol. 2025 Jan 17;15:1456649. doi: 10.3389/fimmu.2024.1456649 (PMC11782277; doi:10.3389/fimmu.2024.1456649)
Supplement: Supplementary file 1 [file DataSheet1.zip › Supplementary Table 5.docx]

**Supplementary Table 5 Univariate and multivariate Cox regression analysis for clinical variables in HNSCC.**

| Characteristics | Total(N) | Univariate analysis | |  | Multivariate analysis | |
| --- | --- | --- | --- | --- | --- | --- |
|  |  | Hazard ratio (95% CI) | P value |  | Hazard ratio (95% CI) | P value |
| Age | 503 |  |  |  |  |  |
| >60 | 256 | Reference |  |  | Reference |  |
| <=60 | 247 | 0.792 (0.605 - 1.037) | 0.090 |  | 0.771 (0.579 - 1.027) | 0.076 |
| Sex | 503 |  |  |  |  |  |
| MALE | 369 | Reference |  |  | Reference |  |
| FEMALE | 134 | 1.316 (0.988 - 1.752) | 0.061 |  | 1.338 (0.990 - 1.808) | 0.058 |
| Race | 487 |  |  |  |  |  |
| BLACK | 47 | Reference |  |  | - | - |
| WHITE | 430 | 0.659 (0.423 - 1.028) | 0.066 |  | - | - |
| ASIAN | 10 | 0.849 (0.292 - 2.468) | 0.763 |  | - | - |
| T | 492 |  |  |  |  |  |
| T1-2 | 179 | Reference |  |  | - | - |
| T3-4 | 313 | 0.791 (0.592 - 1.055) | 0.110 |  | - | - |
| N | 484 |  |  |  |  |  |
| N0 | 241 | Reference |  |  | Reference |  |
| N1-3 | 243 | 0.791 (0.604 - 1.035) | 0.088 |  | 0.930 (0.693 - 1.250) | 0.632 |
| M | 483 |  |  |  |  |  |
| M0 | 478 | Reference |  |  | Reference |  |
| M1 | 5 | 4.819 (1.775 - 13.083) | **0.002** |  | 3.919 (1.414 - 10.861) | **0.009** |
| Stage | 503 |  |  |  |  |  |
| I-II | 105 | Reference |  |  | Reference |  |
| III-IV | 398 | 0.568 (0.394 - 0.821) | **0.003** |  | 0.560 (0.373 - 0.841) | **0.005** |
| Grade | 483 |  |  |  |  |  |
| G1-2 | 362 | Reference |  |  | - | - |
| G3-4 | 121 | 0.942 (0.690 - 1.286) | 0.706 |  | - | - |
| Smoking | 493 |  |  |  |  |  |
| Yes | 380 | Reference |  |  | - | - |
| No | 113 | 0.901 (0.644 - 1.261) | 0.544 |  | - | - |
| Radiation | 183 |  |  |  |  |  |
| NO | 62 | Reference |  |  | - | - |
| YES | 121 | 0.823 (0.492 - 1.378) | 0.459 |  | - | - |
| alcohol | 492 |  |  |  |  |  |
| NO | 159 | Reference |  |  | - | - |
| YES | 333 | 0.954 (0.718 - 1.268) | 0.745 |  | - | - |
| Riskscore | 503 |  |  |  |  |  |
| Low | 252 | Reference |  |  | Reference |  |
| High | 251 | 0.576 (0.438 - 0.757) | **< 0.001** |  | 0.534 (0.403 - 0.709) | **< 0.001** |
