# Supplementary material for: Disulfidptosis-related gene signatures as prognostic biomarkers and predictors of immunotherapy response in HNSCC
Source: Front Immunol. 2025 Jan 17;15:1456649. doi: 10.3389/fimmu.2024.1456649 (PMC11782277; doi:10.3389/fimmu.2024.1456649)
Supplement: Supplementary file 1 [file DataSheet1.zip › Supplementary Table 6.docx]

**Supplementary Table 6. Clinical baseline data of HNSCC.**

| Characteristics | n | Low-riskscore | High-riskscore | P value | t/χ^2^ |
| --- | --- | --- | --- | --- | --- |
| age, n (%) |  |  |  | 0.813 | 0.055759 |
| <60 | 29 | 14 (18.4%) | 15 (19.7%) |  |  |
| ≥60 | 47 | 24 (31.6%) | 23 (30.3%) |  |  |
| gender, n (%) |  |  |  | 0.118 | 2.4429 |
| Female | 20 | 13 (17.1%) | 7 (9.2%) |  |  |
| Male | 56 | 25 (32.9%) | 31 (40.8%) |  |  |
| N stage, n (%) |  |  |  | 0.317 | 3.5294 |
|  |  |  |  |  |  |
| N0 | 10 | 7 (9.2%) | 3 (3.9%) |  |  |
| N1 | 17 | 10 (13.2%) | 7 (9.2%) |  |  |
| N2 | 35 | 16 (21.1%) | 19 (25%) |  |  |
| N3 | 14 | 5 (6.6%) | 9 (11.8%) |  |  |
| distant metastasis, n (%) |  |  |  | 0.304 | 1.0556 |
| M0 | 72 | 37 (48.7%) | 35 (46.1%) |  |  |
| M1 | 4 | 1 (1.3%) | 3 (3.9%) |  |  |
| Clinical stage, n (%) |  |  |  | **0.037** | 4.3429 |
| I/II | 20 | 14 (18.4%) | 6 (7.9%) |  |  |
| III/IV | 56 | 24 (31.6%) | 32 (42.1%) |  |  |
| tumor site, n (%) |  |  |  | 0.072 | 6.9882 |
| Oropharynx | 12 | 4 (5.3%) | 8 (10.5%) |  |  |
| nasopharynx | 37 | 24 (31.6%) | 13 (17.1%) |  |  |
| Cavum Oris | 26 | 10 (13.2%) | 16 (21.1%) |  |  |
| Larynx | 1 | 0 (0%) | 1 (1.3%) |  |  |
| histological grade, n (%) |  |  |  | **0.009** | 9.4291 |
| Well | 29 | 21 (27.6%) | 8 (10.5%) |  |  |
| Moderate | 19 | 7 (9.2%) | 12 (15.8%) |  |  |
| Poor | 28 | 10 (13.2%) | 18(23.7%) |  |  |
| Smoking, n (%) |  |  |  | 0.147 | 2.1046 |
| No |  | 16 (21.1%) | 10 (13.2%) |  |  |
| Yes |  | 22 (28.9%) | 28 (36.8%) |  |  |
| alcohol, n (%) |  |  |  | 0.554 | 0.35023 |
| Yes |  | 30 (39.5%) | 32 (42.1%) |  |  |
| No |  | 8 (10.5%) | 6 (7.9%) |  |  |
| treatment, n (%) |  |  |  | 0.744 | 0.10629 |
| multi |  | 32 (42.1%) | 33 (43.4%) |  |  |
| mono |  | 6 (7.9%) | 5 (6.6%) |  |  |
