# Supplementary material for: Disulfidptosis-related gene signatures as prognostic biomarkers and predictors of immunotherapy response in HNSCC
Source: Front Immunol. 2025 Jan 17;15:1456649. doi: 10.3389/fimmu.2024.1456649 (PMC11782277; doi:10.3389/fimmu.2024.1456649)
Supplement: Supplementary file 2 [file DataSheet2.zip › Supplementary Figure(1).docx]

**Supplementary Figure 1:** Correlation analysis of genetic alterations in DRGs. (A) The distribution of mutation types in the first 10 DRGs in HNSCC. (B-D) Variant classification, variant type, SNV class. (E) Mutation burden per sample. (F) Variant classification summary. (G) The top 10 mutated genes in HNSCC. SNP, single nucleotide polymorphism. (H) The relationship between CNV, methylation, and DRGs expression. Red indicates positive correlation; blue indicates negative correlation. The deeper the color, the higher the correlation index. Bubble size represents the FDR (False Discovery Rate). (I) The correlation between DRGs CNV, methylation and survival rates (including DFI, DSS, OS, PFS) in HNSCC patients. (J) CNV pie chart distribution. (K) Hete CNV map and homo CNV profile of 24 DRGs in HNSCC cohort. SNP, single nucleotide polymorphism; SNV, single nucleotide variation; CNV, copy number changes.

**Supplementary Figure 2:** Construction of a PFS, DSS model for HNSCC prognosis with the help of the four prognostic DRGs. (A, D) Distribution of risk score, survival status, and expression of prognostic DRGs in HNSCC patients. (B) PFS curve of HNSCC patients in high/low-risk groups. (C) Time-dependent ROC curve for 1-, 3-, and 5-year PFS for DRGs. (E) DSS curve of HNSCC patients in high/low-risk groups. (F) Time-dependent ROC curve for 1-, 3-, and 5-year DSS for DRGs.

**Supplementary Figure 3:** The survival curve of high-risk and low-risk patients in different subgroups of HNSCC, including Age > 60 (A), Female (B), male (C), White (D), Grade 1-2 (E), Grade 3-4 (F), Stage III-IV (G), M0 Status (H), N0 Status (I), N1-3 Status (J), T3-4 Status (K), Neoadjuvant N0 (L), Smoking Yes (M).

**Supplementary Figure 4:** The survival curve of high-risk and low-risk patients in different subgroups of HNSCC, including Age <=60 (A), Asian+Black (B), Stage I-II (C), Neoadjuvant Yes (D), Radiation N0 (E), Radiation Yes (F), Smoking N0 (G), T1-2 Status (H).

**Supplementary Figure 5:** Construction of a predictive nomogram (PFS). (A) Nomogram for predicting 1-, 3-, and 5-year PFS of HNSCC patients. (B) ROC curves for predicting 1-, 3-, and 5-year PFS in the TCGA. (C) Time-dependent AUC curve shows the nomogram to predict PFS performance. (D) Calibration curve of PFS nomogram model in the discovery group. The diagonal dotted line represents the ideal nomogram. (E) DCA curves for the nomogram.

**Supplementary Figure 6:** Construction of a predictive nomogram (DSS). (A) Nomogram for predicting 1-, 3-, and 5-year DSS of HNSCC patients. (B) ROC curves for predicting 1-, 3-, and 5-year DSS. (C) Time-dependent AUC curve shows the nomogram to predict DSS performance in the TCGA. (D) Calibration curve of DSS nomogram model in the discovery group in the TCGA. The diagonal dotted line represents the ideal nomogram. (E) DCA curves for the nomogram.

**Supplementary Figure 7:** Relationship between the expression level of DRGs in the tumor microenvironment and immune infiltration. (A-E) Comparison of immune scores between C1 and C2 subtypes in TCGA. (A) CIBERSORT, (B) TIMER, (C) EPIC, (D) xCell, (E) MCPcounter.

**Supplementary Figure 8:** Relationship between Riskscore and immune scores through different algorithms. (A) CIBERSORT, (B) TIMER, (C) EPIC, (D) xCell, (E) MCPcounter.

**Supplementary Figure 9:** Relationship between DRGs expression and immune infiltration in HNSCC. (A) Differences in immune cell infiltration between the high and low expression groups of the four prognostic DRGs in HNSCC. (B) Differences in immune cell between high and low riskscore group. (C) Correlation between the four prognostic DRGs in HNSCC and tumor immune cell infiltration. (D) Relationship between the abundance of immune cells in HNSCC and the expression levels of four prognostic DRGs in TIMER database.

**Supplementary Figure 10:** The association between the prognostic DRGs and pan-RNA epigenetic modification-related genes. (A) Differences in m6A, m5C, m1A, and m7G between the high and low risk group in HNSCC. (B) The correlation between three prognostic DRGs and m6A, m5C, m1A, m7G-related genes in the TCGA-HNSCC cohort. (C) EIF4E, IGF2BP3, FTO, IFIT5, IGF2BP1, LARP1, NCBP2L, NUDT10, and NUDT11 survival curve.

**Supplementary Figure 11:** The DNA methylation analysis of the four prognostic DRGs in HNSCC. (A) Correlation between the DRGs mRNA expression and DNA methylation levels in HNSCC. (B) Promoter methylation levels of NUBPL in HNSCC based on different clinical and pathological features, **P<0.01; ***P<0.001. (C) Heatmap of DNA methylation of DRGs genes CpG sites in HNSCC obtained using MethSurv database. (D, E) Prognostic value of DNA methylation at different CpG sites of DRGs in HNSCC patients.

**Supplementary Figure 12:** Identification of the most potential miRNAs associated with the prognosis of HNSCC. (A) Prediction of the potential miRNAs of the four prognostic DRGs through ENCORI, miRTarBase, RNA22, RNAInter, and miRWalk databases. (B) Construction of a potential miRNA gene network using the Cytoscape software. (C) The prognostic value of miRNAs (hsa-let-7c-5p, hsa-miR-181c-5p, and hsa-miR-23b-5p) in HNSCC. (D) The expression level of miRNAs (hsa-let-7c-5p, hsa-miR-181c-5p, and hsa-miR-23b-5p) in HNSCC.

**Supplementary Figure 13:** Significant negative correlations between 4 pairs of ACTB-miRNA and 6 pairs of DSTN-miRNA
